# Metal-fluorouracil networks with disruption of mitochondrion enhanced ferroptosis for synergistic immune activation

**DOI:** 10.7150/thno.75323

**Published:** 2022-08-21

**Authors:** Lingling Lei, Zhe Dong, Li Xu, Fengrui Yang, Baoli Yin, Youjuan Wang, Renye Yue, Guoqiang Guan, Juntao Xu, Guosheng Song, Xiao-bing Zhang

**Affiliations:** State Key Laboratory of Chemo/Bio-Sensing and Chemometrics, College of Chemistry and Chemical Engineering, Hunan University, Changsha 410082, P. R. China.

**Keywords:** Immunotherapy, ferroptosis, fluorouracil, ^19^F MR imaging, cancer theranostics

## Abstract

**Rationale:** Ferroptosis drugs inducing cancer immunogenic cell death (ICD) have shown the potential of immunotherapy *in vivo*. However, the current ferroptosis drugs usually induce the insufficient immune response because of the low ROS generation efficiency.

**Methods:** Herein, we design zinc-fluorouracil metallodrug networks (Zn-Fu MNs), by coordinating Zn and Fu via facile one-pot preparation, to inactivate mitochondrial electron transport for enhanced ROS production and immune activation.

**Results:** Zn-Fu MNs can be responsive toward acidity and adenosine triphosphate (ATP) with the release of Fu and Zn^2+^, during which Zn^2+^ can induce mitochondrion disruption to produce ROS, resulting in ferroptosis of cancer cells and 5-Fu interferes with DNA synthesis in nuclei with ^19^F-MRI signal to be switched on for correlating drug release. With the synergistic effect of DNA damage and ferroptosis, the cancer cells are forced to promote ICD. Thereby, Zn-Fu MNs exhibit the excellent immune response without any other antigens loading. As a result, the infiltration of T cells within tumor and activation of immune cells in spleen have been greatly enhanced.

**Conclusions:** Combined DNA damage and ferroptosis, Zn-Fu MNs induce the violent emission of tumor associated antigens within cancer cells which will sensitize naive dendritic cells and promote the activation and recruitment of cytotoxic T lymphocytes to exterminate cancer cells. Therefore, the obtained Zn-Fu MNs as ferroptosis inducers can effectively remodel immunosuppressive tumor microenvironment and activate antitumor immune reaction.

## Introduction

Currently, immunotherapy has been considered as a promising method for cancer treatment, which works by stimulating the innate immune response of body to fight against tumor cells 1. However, the complex of tumor microenvironment (TME) and silent innate immune system result in the boundedness of most immunotherapeutic strategies for cancer therapy in clinical applications. Moreover, tumor cells utilize immune escape to protect themselves from being cleared by the immune system 2-4. Therefore, the immediate dilemma of immunotherapy is to reverse tumor immune and enhance anti-tumor immunity. Fortunately, immunogenic cell death (ICD) as a new tumor death mode have been developed to immunotherapy, which would expose large number of antigens to activate immune system 5-7. Some chemotherapeutic drugs, such as anthracyclines and DNA damage drugs, have been reported to cause immunogenic cell death (ICD) 8,9. However, the immune activation induced by drug injury alone is not effective. In addition, some physical therapies, such as radiotherapy, extracorporeal photochemotherapy, photodynamic therapy (PDT), near-infrared light immunotherapy, etc., have been reported that can induce ICD activation and immune activation effect 10-14. Nevertheless, the overproduction of immunogenicity can lead to uncontrollable systemic inflammation and severe complications of multiple organ dysfunction syndrome, thereby inducing no satisfied immunotherapy. Therefore, it is of great significance to develop novel strategy to effectively induce immune response and enhance anti-tumor response.

Ferroptosis, a new type of programmed/regulated cell death which is different from classic cell death pathways like necrosis or apoptosis, is mediated by the production of iron-dependent reactive oxygen species, the inactivation of glutathione peroxidase 4 (GPX4) and further accumulation of lipid peroxides 11,15. Significantly, ferroptosis cells show necrotic morphological changes, including cytoplasmic swelling, chromatin condensation, membrane burst and damage. Moreover, recent studies suggested that the ferroptosis can spread out between cell populations in a wavy like manner, leading to cell death in a spatiotemporal mode 16. Based on the unique ways of death, the ferroptosis has been developed to effectively produce immunogenicity then inhibit tumor growth 17,18. Recently, the commonly used ferroptosis agents, such as MnO_x_, Fe_3_O_4_, single-crystal Fe (0), produced oxidative stress in cancer cells based on Fenton reaction by decomposition endogenous hydrogen peroxide (H_2_O_2_) to generate ·OH 11,18-21. However, the slightly acidic tumor microenvironment leads to the decrease of iron-based Fenton reaction efficiency. What's more, the insufficient endogenous substances have led to the inefficient oxidation reaction that could not induce an effective immune response. Therefore, it is desirable to find more superior ferroptosis inducer with effective antitumor immune.

Fluorouracil (5-Fu or Fu) is an organic molecular drug that has been widely used in clinical practice 22-23. Although Fu induced chemotherapy has been demonstrated to induce ICD in tumor cells, the unsatisfactory immune response and inevitable toxic side effects were observed *in vivo*, because of the drug resistance and low selectivity 23-26. Recently, several carriers, such as graphene 27, covalent organic framework 28, selenium nanoparticles 29, or gold nanoparticles 30 have been used for loading 5-Fu to improve its tumor targeting. However, the drug delivery efficiency of these vectors was not ideal for further applications. On the other hand, ^19^F magnetic resonance imaging (MRI) is an ideal approach as a biological imaging technology because of its negligible background and unlimited tissue penetration depth 31. Most of the current fluorine imaging probes are perfluorocarbons or long-chain polymers, which lead to the complexity of synthesis and unstable ^19^F signal output 31-33. 5-Fu exhibits a potential for ^19^F MR imaging due to the internal fluorine atom 34,35. However, the low fluorine content in current vectors of Fu limit theirs further application in the field of ^19^F-MRI imaging. Thus, it is desirable to de novo design an efficient delivery system for 5-Fu, so as to strengthen immunogenic effect of Fu and ^19^F-MRI.

**Scheme 1:**
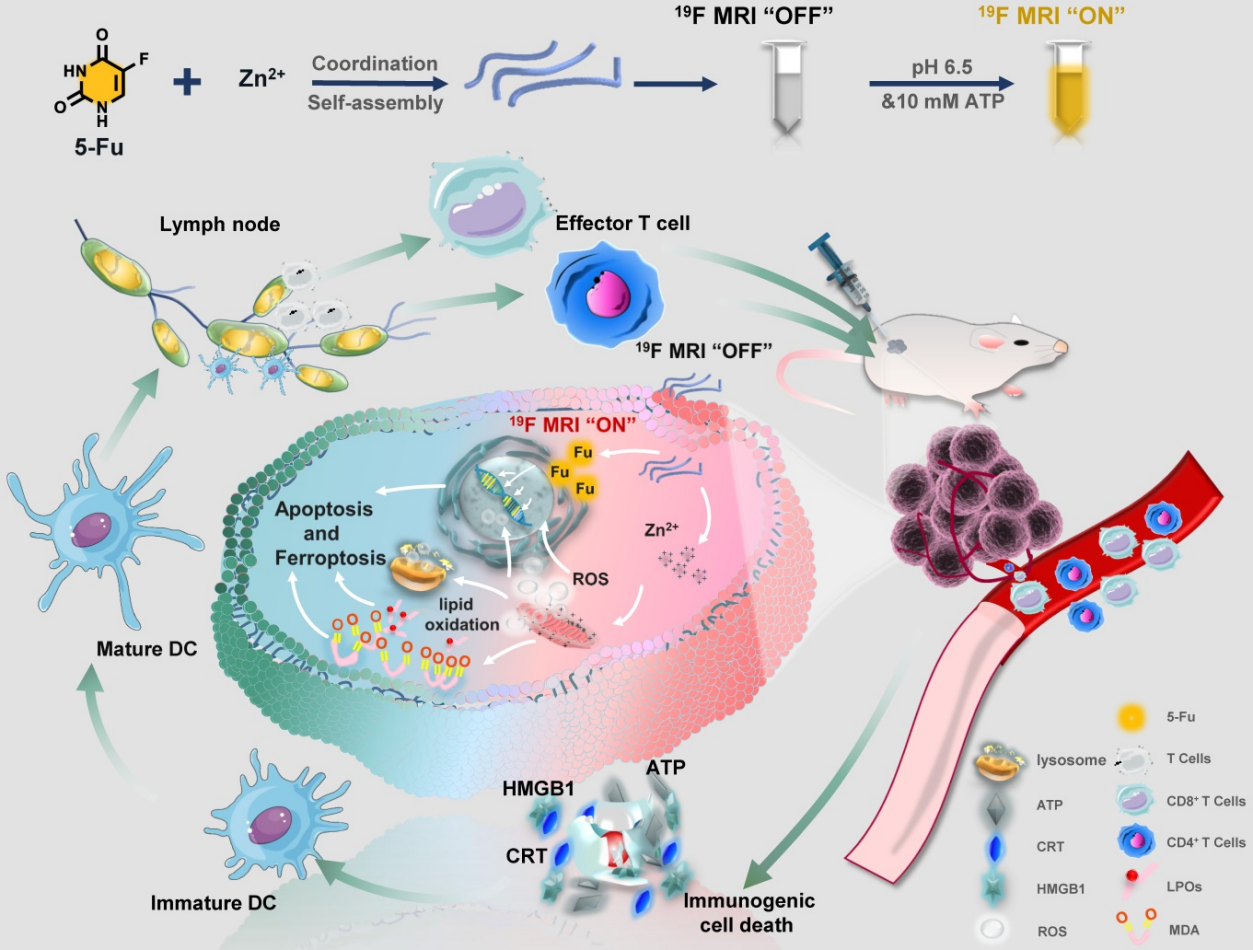
Schematic illustration of Zinc-Fluorouracil metallodrug networks can remold immunosuppressive TME and induce immune activation through synergistic effect of Fu-induced DNA damages and Zn-induced mitochondrial dysfunction.

Herein, a facile “one-pot” method was developed for constructing zinc-fluorouracil metallodrug networks (Zn-Fu MNs) with coordination-driven self-assembly, which exhibited the high-drug loading efficiency (40.93wt%). The obtained Zn-Fu MNs could act as tumor microenvironment-responsive (acidity and adenosine triphosphate (ATP) ferroptosis agents through inhibiting the electron transport chain, resulting in a distinct mitochondrial reactive oxygen species (ROS) production (Scheme 1). Notably, dual damages induced by Fu-mediated DNA transcription inhibition and Zn-induced ferroptosis led to a violent ICD burst in the cancer cells and then the large amount of antigens release. As a result, a large number of T cells were invaded within tumor and immune cells were activated in the spleen, which will effectively counter tumor growth. Besides, with the high Fu loading efficiency, Zn-Fu MNs achieved the satisfied fluorine content for ^19^F-MRI imaging. Due to the inflexible skeleton and aggregation of F element within Zn-Fu MNs, the ^19^F signal was completely “quenched” in the initial state. In the presence of both acidity and ATP, Zn-Fu MNs were disassembled to release Fu drug and Zn^2+^ and showed “turn on” ^19^F-MRI, promising for indicating drug release and ferroptosis *in vivo*.

## Results

### Synthesis and characterization of Zn-Fu MNs

**Figure 1:**
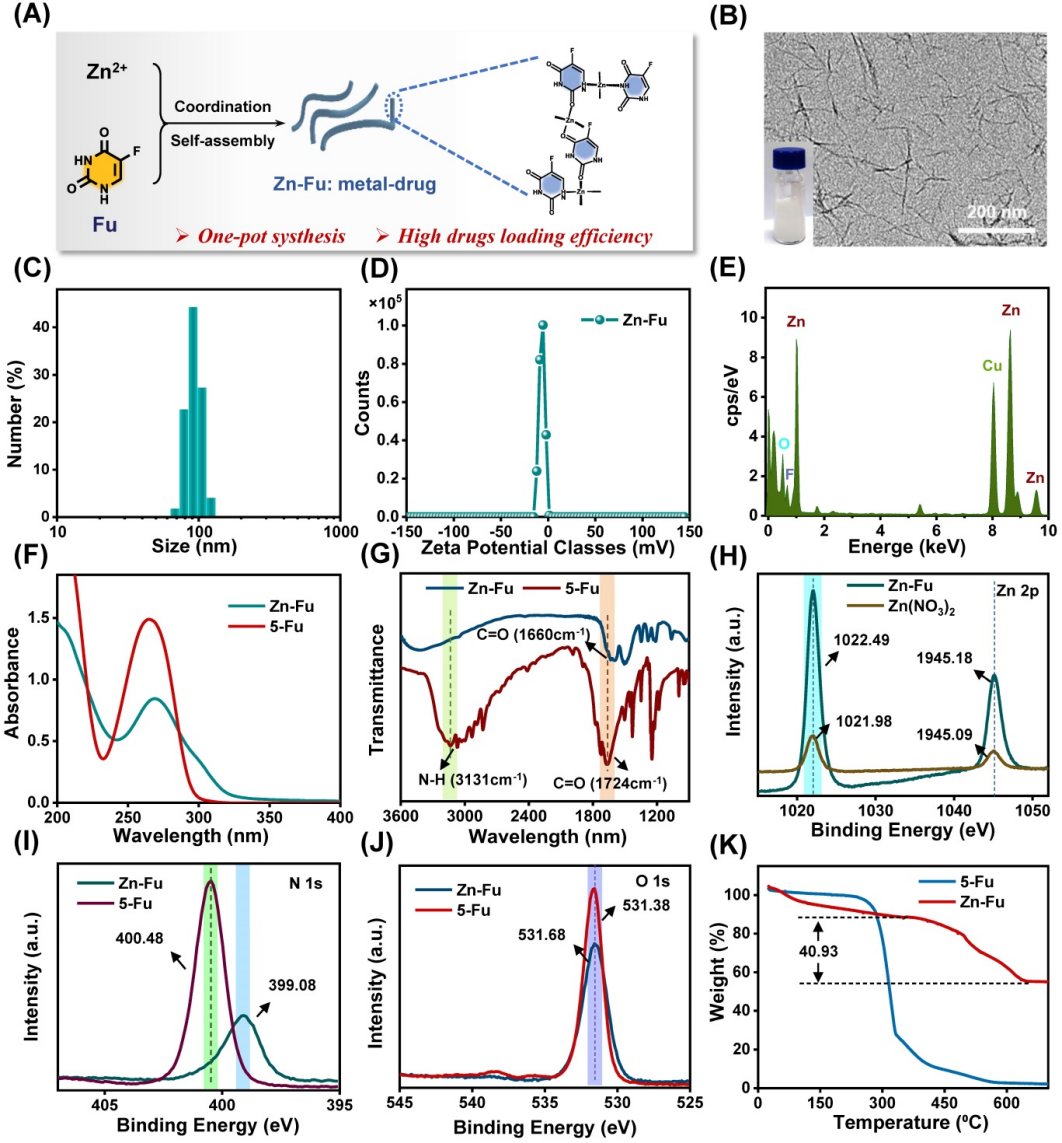
**Synthesis and Characterization of Zn-Fu MNs. (A)** Illustration of composition of Zn-Fu MNs. **(B)** TEM image of Zn-Fu MNs. **(C)** DLS profile of Zn-Fu MNs.** (D)** Zeta potential of Zn-Fu MNs. **(E)** EDS spectra of Zn-Fu MNs. **(F)** UV-Vis spectra of Zn-Fu MNs and 5-Fu. **(G)** FT-IR spectra of 5-Fu and Zn-Fu MNs nanodrugs. **(H)** XPS spectra of Zn 2p in Zn(NO_3_)_2_ and Zn-Fu MNs nanodrugs. **(I)** XPS spectra of N 1s in 5-Fu and Zn-Fu MNs nanodrugs. **(J)** XPS spectra of O 1s in 5-Fu and Zn-Fu MNs nanodrugs. **(K)** Thermogravimetric curves of 5-Fu and Zn-Fu MNs.

Using a coordinated-driven self-assembly strategy, Zn-Fu MNs was obtained by simply mixed fluorouracil and zinc nitrate in one pot under sonication (Figure 1A). Then systematical characterizations of Zn-Fu MNs were carried out using transmission electron microscopy (TEM), dynamic light scattering (DLS), ultraviolet-visible (UV), Fourier transform infrared (FTIR), X-ray photoelectron spectroscopy (XPS) and thermo-gravimetric analysis (TGA). The TEM and DLS showed Zn-Fu MNs with threadiness morphology (Figure 1B), average dynamic size and zeta potential about 100 nm and -6.96 mV, respectively (Figures 1C, 1D). The good combination of Zn, O and F in Zn-Fu MNs was demonstrated by energy dispersive X-ray spectroscopy (EDS) (Figure 1E). UV spectra showed Zn-Fu MNs with the typical spectrum peaks of Fu and slight red sight (Figure 1F). According to the analysis of FTIR spectroscopy, a broad absorption peak at 3131 cm^-1^ was attributed to N-H stretching vibration in Fu and which disappeared in Zn-Fu MNs (Figure 1G), indicating the successful coordination between Zn and N 36. Besides, the C=O stretching vibration of Fu appeared at 1724cm^-1^, which shifted to 1660 cm^-1^ in Zn-Fu MNs, indicating the coordination between Zn and O. In XPS, the element absorption peak of Zn, N, O and C appeared in Zn-Fu MNs (Figure S1), while the slight peaks deviation of Zn2p (from 1021.98 eV shift to 1022.494 eV) (Figure 1H), N1s (from 400.48 eV shift to 399.08 eV) (Figure 1I) and O1s (from 531.68 eV to 531.38 eV) (Figure 1J) were observed, confirming the composition of Zn-Fu MNs was driven by the coordination of divalent zinc ions with N and O of Fu molecules 36. From TGA measurement, the mass percentage was determined to be 40.93% for Fu in Zn-Fu MNs, indicating the high drug loading capability (Figure 1K). Through the one-pot synthesis method, the total mass of Zn-Fu MNs was more than 160 mg. The constant particle size at different times proved the stability of Zn-Fu MNs in phosphate buffered saline (PBS) (Figure S2).

### ^19^F MR imaging (^19^F-MRI) correlated drug release

**Figure 2:**
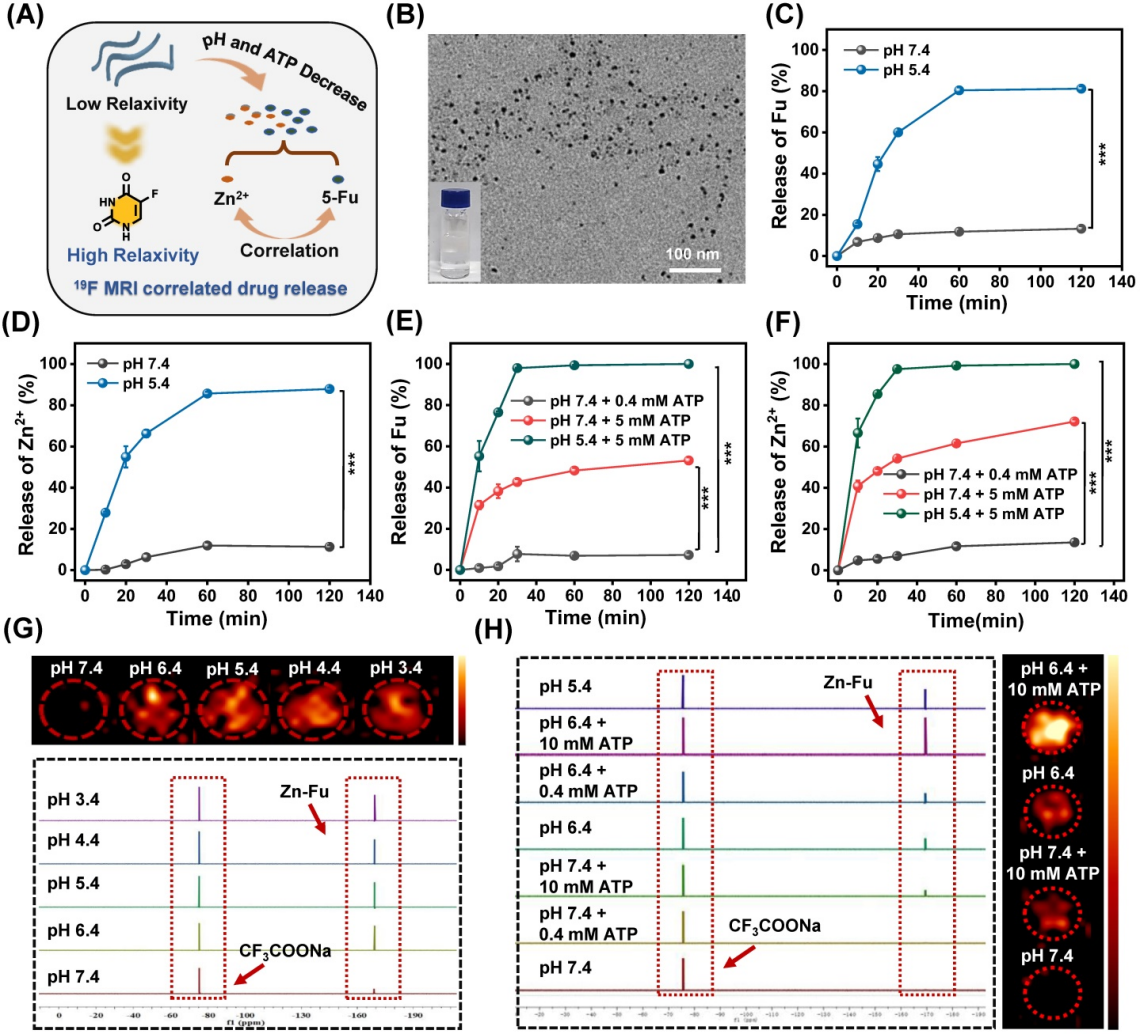
**^19^F-MRI Correlated Drug Release. (A)** Illustration of pH and ATP triggered release of Fu and Zn^2+^, correlated with ^19^F-MRI. **(B)** TEM image of Zn-Fu MNs, after treatment in acidic condition. **(C)** The release profile of 5-Fu at pH 7.4 or 5.4. **(D)** The release profile of Zn at pH 7.4 or 5.4. **(E)** The release profile of 5-Fu at pH (7.4 or 5.4) and ATP (0.4 or 5 mM). **(F)** The release profile of Zn^2+^ at pH (7.4 or 5.4) and ATP (0.4 or 5 mM). Error bars denote the standard deviation (n = 3). **(G)** ^19^F NMR spectra of Zn-Fu MNs incubated in different pH condition and corresponding ^19^F-MRI images, using CF_3_COONa as reference. **(H)** ^19^F NMR spectra of Zn-Fu MNs treated in different pH condition + ATP, and ^19^F MR images. P-values were determined using one-way analysis of variance (ANOVA): *p < 0.05, **p < 0.01, ***p < 0.001.

Tumor microenvironment is featured with low pH 37-38 and cytoplasm has high concentrations of ATP (1-10 mM) 39-40. Drive by the weak coordination between Zn and N, we explored the disintegration conditions of Zn-Fu MNs. Interestingly, we found that due to the easy protonation of nitrogen 36 and the Zn coordination competitive between ATP and Fu 41, Zn-Fu MNs were able to be decomposed in the presence of acidity and ATP with release of Fu and Zn^2+^ synchronously (Figure 2A). At first, we tested the morphology change of Zn-Fu MNs responding toward acidity. After incubation in acidic condition, the TEM image (Figure 2B) showed that the threadiness morphology of original Zn-Fu MNs was converted to fine particles (about 5 nm). Furthermore, DLS also proved the size of original Zn-Fu MNs was converted from 92 nm to 5 nm, indicating the acidity triggered structure disintegration for Zn-Fu MNs (Figure S3). In addition, the reversal zeta potential (from -6.96 mV to 20.12 mV) in different pH also proved the acid sensitivity of Zn-Fu MNs (Figure S4). Next, we explored the release dynamics of Fu and Zn^2+^ from Zn-Fu MNs, via incubating Zn-Fu MNs in the presence of acidity or/and ATP, and collecting the supernatant of Zn-Fu MNs solution for measuring Fu and Zn by the standard curves of UV-Vis absorption and ICP-MS, respectively. In the presence of pH 7.4 and/or low concentration ATP (0.4 mM), the Fu release was less than 15%, proving the good stability of Zn-Fu MNs in the environment of neutral and low concentration ATP. As contrast, at pH 5.4, the Fu release was greatly increased to 80% after 120 min incubation. Moreover, the drug release could be further boosted to 100 %, upon supplement of ATP (5 mM) in pH 5.4. Besides, the supplement of ATP (5 mM) in pH 7.4 was able to promote the drug release (47%), in contrast to that at pH 7.4 with low concentration ATP (0.4 mM) (Figures 2C-2D). As expected, the release profile of Zn from Zn-Fu MNs showed the similar tendency with that of Fu (Figures 2E-2F). Therefore, these results confirmed the acidity and ATP triggered structure collapse of Zn-Fu MNs and the burst release of Zn/Fu.

On account of the spin-spin relaxations and molecular mobility restriction, ^19^F signal of Zn-Fu MNs was completely in “quenched” 32,42. Upon exposure to the TME (acidity and high concentration of ATP) 40 the coordination bonds in Zn-Fu MNs were broken, leading to weak intermolecular interactions and higher molecular mobility, which would expect to bring about the recovered ^19^F signal. Thereby, we systematically investigated the correlation between the drug release and ^19^F signal “turn on”. The Zn-Fu MNs were incubated in PBS buffer solutions (pH = 7.4, 6.4, 5.4, 4.4 and 3.4) for ^19^F NMR analysis, taking sodium trifluoroacetate as reference (Figure 2G). From ^19^F NMR spectra, no ^19^F NMR signal was detected in the neutral environment (pH 7.4), while the ^19^F NMR signal was sharply increased (5 times) in slightly acidic environment (pH 6.4). Meanwhile, phantom imaging was carried out to explore ^19^F-MRI contrast, via 7.0 T MRI scanner (Bruker). Zn-Fu MNs displayed negligible ^19^F-MRI signals in pH 7.4, while strong ^19^F MR imaging contrast was observed in acidic condition (from pH 6.4 to pH 3.4), indicating the acidity triggered ^19^F MR imaging ability.

Furthermore, we explored the co-activation effect of pH and ATP on ^19^F NMR signal, via incubating Zn-Fu MNs in pH (7.4 or 5.4) and various concentration of ATP (0.4 mM or 10 mM), during which the neutral buffer solution (pH 7.4 + 0.4 mM of ATP) simulated the normal tissue and the slight acid and high concentration of ATP (pH 6.4 + 10 mM of ATP) represented TME (Figure 2H). From ^19^F NMR spectra, no ^19^F NMR was found from Zn-Fu MNs in the presence of pH 7.4 and/or 0.4 mM of ATP, suggesting the “off state” ^19^F NMR in the simulated normal tissue environment. Surprisingly, significant enhancement of ^19^F NMR signal was detected for Zn-Fu MNs in the presence of pH 5.4 + 10 mM of ATP, which is about 13.3 folds of that in pH 7.4 + 0.4 mM of ATP, indicating the potential of TME triggered the burst increase of ^19^F NMR signal. Furthermore, phantom imaging collected from 7.0 T MRI scanner showed the strongest “hot spot” in ^19^F MR images for Zn-Fu MNs in pH 6.4+10 mM ATP, compared with no obvious ^19^F-MRI contrast in pH 7.4, verifying the co-activation effect of acidity and ATP for “turn on” ^19^F MR imaging contrast, which was consistent with the acidity + ATP triggered burst release of Fu.

### Zn-Fu MNs mediated ferroptosis *in vitro*

**Figure 3:**
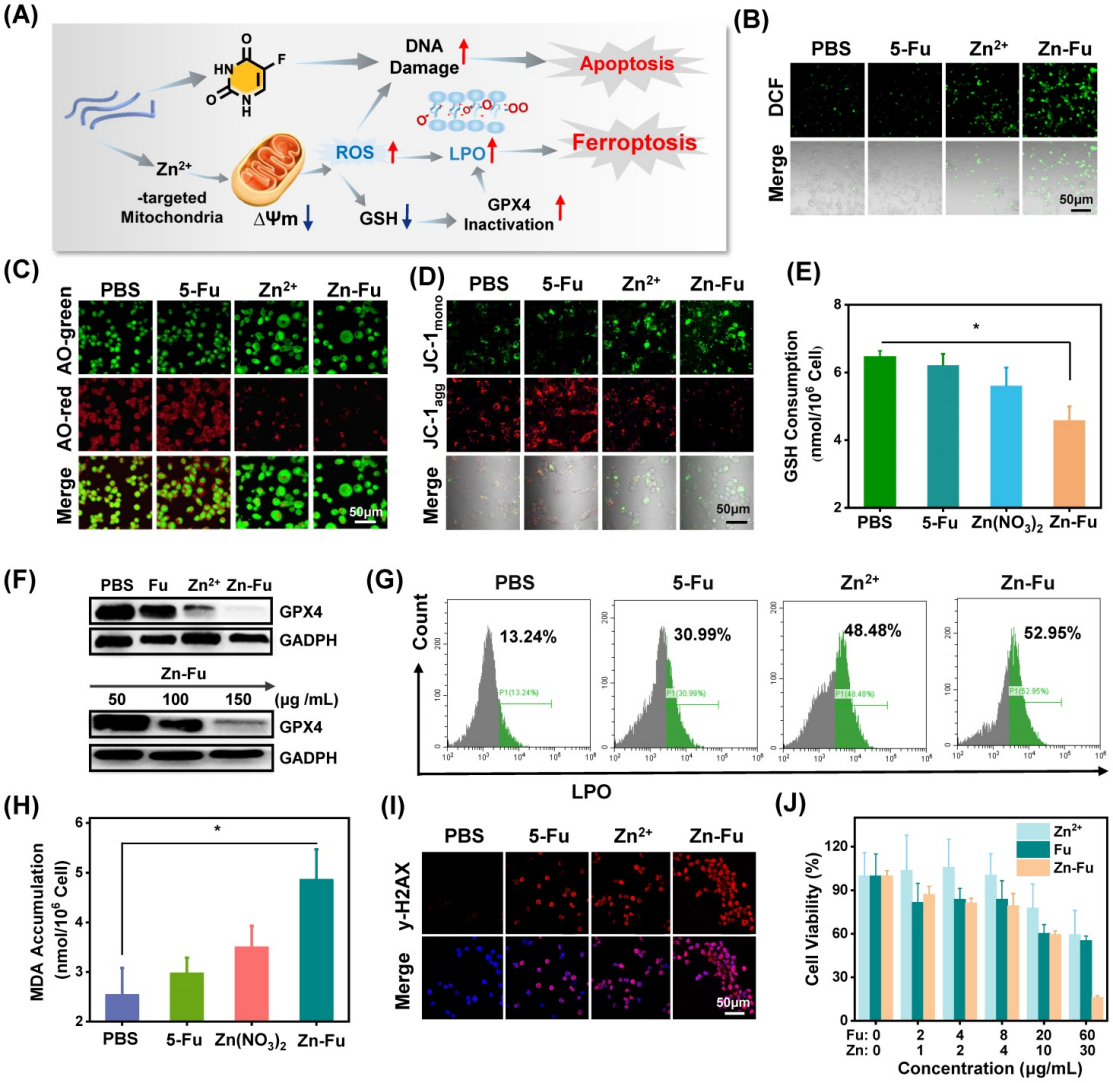
**The Ferroptosis Effect of Zn-Fu MNs. (A)** Illustration of Zn-Fu MNs mediated ferroptosis and Fu mediated apoptosis. **(B)** Intracellular ROS of cancer cells after various treatment, using DCFH-DA as probe. **(C)** Acridine (AO) staining of cancer cells after various treatment. The green fluorescence is free deprotonated AO in the envelope while the red fluorescence that is emitted by protonated oligomeric AO characterizes the membrane integrity of endo/lysosomes. **(D)** Mitochondrial membrane potential of cancer cells after various treatment, using JC-1 probe, the red fluorescence that is emitted by aggregated JC-1 characterizes a high membrane potential while green fluorescence emitted by JC-1 monomer characterizes low membrane potential. **(E)** Intracellular GSH content of cancer cells after various treatment, using GSH Assay Kit. **(F)** Western blots of glutathione-dependent peroxidases 4 (GPX4) of cancer cells after various treatment. **(G)** LPO level of cancer cells after various treatment, using Liperfluo as probe for flow cytometry. **(H)** MDA content in cancer cells after various treatment, measured by MDA Assay Kit. **(I)** γ-H_2_AX stained cancer cells after various treatment. Red indicated DNA damage. **(J)** Cell viability of CT26 cancer cells incubated with different concentrations of Zn-Fu MNs for 24 h. P-values were determined using one-way ANOVA: *p < 0.05. Error bars denote the standard deviation (n = 3).

The burst release of Zn^2+^ would lead to the cellular accumulation of Zn and increase the production of mitochondrial O_2_·^-^ by inhibiting the electron transport chain of mitochondria 43-46. As a result, large amount of reactive oxygen species (ROS) would be produced, resulting in the break of redox balance within cancer cells 47-49, elimination of overexpressed glutathione (GSH) and the inactivation of glutathione peroxidase 4 (GPX4) protein. These would aggravate the excessive accumulation of lipid oxidation (LPOs) and trigger cancer cells ferroptosis (Figure 3A) 50-51. To validate the above proposed process, the intracellular concentration of Zn^2+^ for Zn-Fu MNs treatment cancer cells was detected by ICP-MS, which showed the distinct increased Zn^2+^ within Zn-Fu MNs treatment cells compared with PBS group, proving the Zn-Fu MNs entered the cells, successfully (Figure S5). Subsequently, we tested the cellular ROS production via incubating CT26 cells with Zn^2+^, Fu and Zn-Fu MNs, respectively, using 2',7'-dichlorofluorescin diacetate (DCFH-DA) as ROS indicator (Figure 3B) 51. From confocal fluorescent images, obvious fluorescence in green channel was observed in Zn^2+^ treatment cells, indicating the intracellular ROS generation induced by Zn^2+^. Moreover, the intracellular ROS generation was further boosted after incubation with Zn-Fu MNs, compared with Zn treated group. Besides, we quantified the intracellular ROS production by flow cytometry, and found the similar tendency with confocal imaging results, demonstrating Zn-Fu MNs induced the oxidative stress toward cancer cells (Figure S6).

Because ROS may lead to the oxidation and rupture of endosomal membrane, we studied the membrane integrity via acridine orange (AO) staining (Figure 3C) 11. From the confocal images, the reduced red dots were observed for Zn^2+^ or Zn-Fu MNs treated cancer cells, confirming the loss integrity of endosomal membrane. Next, we tested the change of mitochondrial membrane potential induced by Zn-Fu MNs toward CT26 cancer cells, using the fluorescent probe of JC-1 19. Specifically, the red fluorescence intensity of aggregated JC-1 demonstrated the normal membrane potential, while green fluorescence of monomer demonstrated the destructive membrane potential. From confocal images (Figure 3D), those cells treated with Zn-Fu MNs or Zn^2+^ showed the increased green fluorescence and reduced red fluorescence, compared with that treated with PBS or Fu, verifying the mitochondrial damage induced by Zn-Fu MNs or Zn^2+^.

Furthermore, we measured the GSH concentration within cancer cell via GSH assay kit (Figure 3E) 18. The intracellular concentration of GSH for Zn-Fu MNs or Zn^2+^ treatment cancer cells showed the distinct down-regulation compared with PBS group, meaning the destroyed redox balance within cancer cells induced by Zn-Fu MNs or Zn^2+^.

Considering the eliminated intracellular GSH, we measured the down regulation of GPX4 protein via Western blots, using the glyceraldehyde-3-phosphate dehydrogenase (GADPH) protein as internal reference for the identification of GPX4 protein activity (Figure 3F, Figure S7) 50. We found that a significant decrease of GPX4 activity was observed for Zn^2+^ or Zn-Fu MNs treated cancer cells, in contrast to PBS or Fu group, suggesting the great inactivation of GPX4, induced by Zn^2+^ or Zn-Fu MNs. Moreover, as the concentration of Zn-Fu MNs increasing from 50 to 150 μg/mL, the signal intensity of GPX4 protein was gradually decreased, validating the concentration of Zn-Fu MNs influencing on GPX4 protein activity.

After that, we examined the level of lipid oxidation for cancer cells after various treatments, with Liperfluo probe by flow cytometry analysis (Figure 3G) 11. We found that Zn^2+^ or Zn-Fu MNs treated groups showed the higher level of LPOs, owing to the ROS production and GPX4 inactivation within those cancer cells induced by free Zn^2+^ or Zn-Fu MNs. Meanwhile, the slight up-regulation of LPOs were also found for Fu treated cancer cells, which was attributed from the DNA damage caused by Fu and thereby the elevated oxidative stress. Then, the generation of malondialdehyde (MDA) was considered as one of the main products of LPOs which was also verified using MDA assay kit (Figure 3H) 52. We observed the elevated content of MDA within cancer cells treated with Zn-Fu MNs, compared with PBS group, further confirming the severe LPOs for Zn-Fu MNs treated cancer cells.

5-Fu drugs as chemotherapeutic drug can disturb DNA transcription and Zinc is able to disrupt the redox balance, which would act the synergistically damage to DNA in tumor cells. Therefore, we investigated the DNA damage induced by Zn^2+^, Fu or Zn-Fu MNs, via γ-H_2_AX staining (Figure 3I) 53. From confocal images, those cancer cells treated with only Zn^2+^ or Fu showed the obvious γ-H_2_AX stained fluorescence signals, in contrast with PBS group, which indicated the disturbed DNA transcription. Furthermore, Zn-Fu MNs induced the higher level of DNA damage than other group, owing the synergistic effect of Zn^2+^ and Fu. Inspired by the efficient ferroptosis and chemotherapeutics mediated by Zn-Fu MNs, we explored the anticancer effect of Zn-Fu MNs toward CT26 cells via co-incubating with various concentrations of Fu, Zn^2+^ or Zn-Fu MNs, respectively, and detecting by standard MTT assay (Figure 3J) 54. As the concentration increasing, the cellular viability of cancer cells treated with Zn-Fu MNs was gradually depressed, indicating concentration-dependent anticancer effect. Furthermore, Zn-Fu MNs exhibited more cellular apoptosis and necrosis than other group (Figure S8), which confirmed the excellent anticancer activity of Zn-Fu MNs. In addition, we further investigated the proportion of apoptosis and ferroptosis induced by Zn-Fu MNs using a LPO probe Liperfluo and Annexin V-mCherry apoptosis detection kit. As a result, we found that Zn-Fu MNs treated CT26 cells showed the higher proportion of ferroptosis and apoptotic than PBS group (Figure S9) 55.

### *In vivo* immune effect

**Figure 4:**
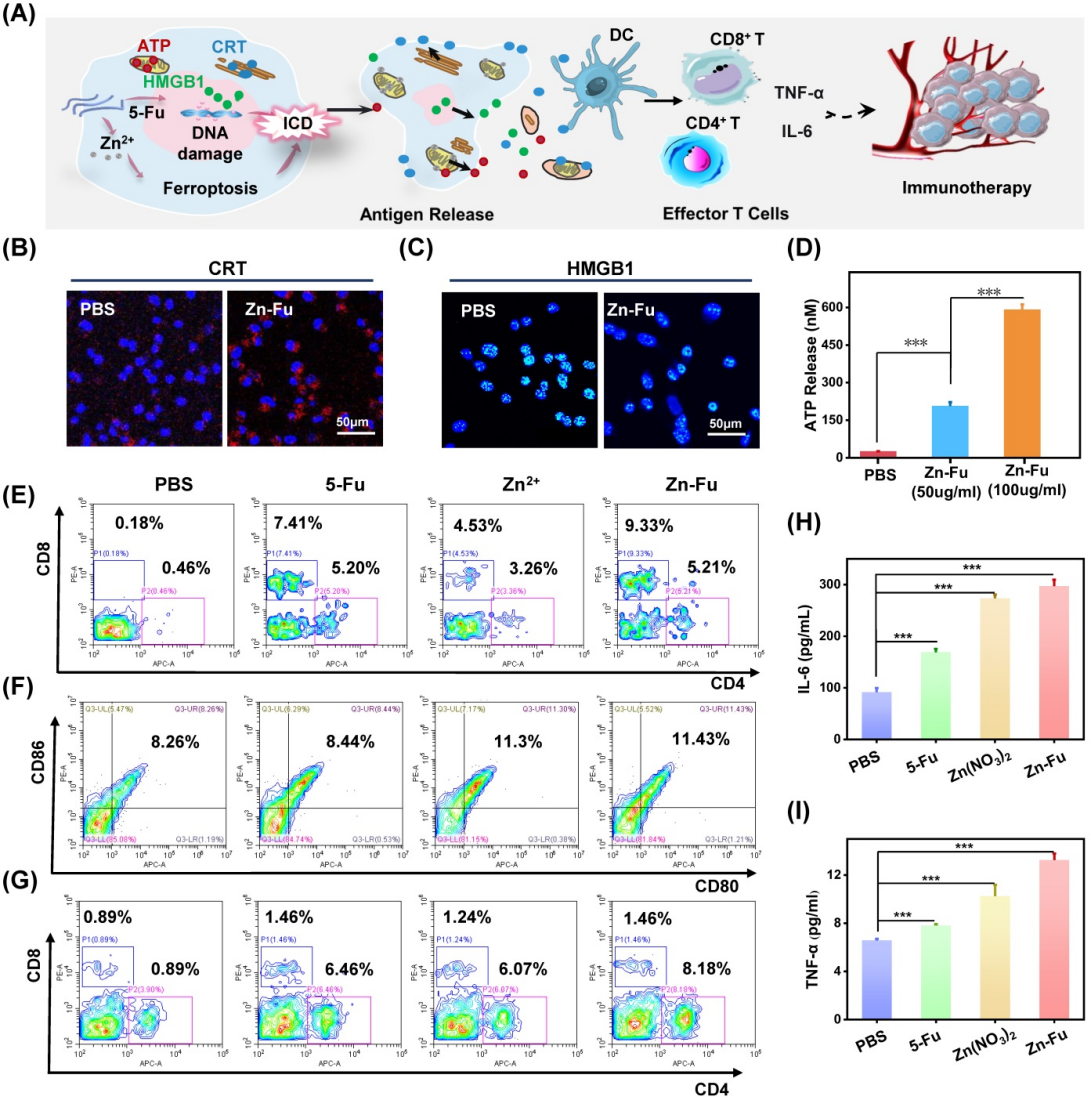
**Zn-Fu MNs Mediated Immunogenic Cell Death (ICD) and Immune Response. (A)** Illustration of Zn-Fu MNs mediated immune activation. **(B)** Confocal images of cancer cells, stained with CRT or **(C)** HMGB1, treated by Zn-Fu MNs or not, for visualizing CRT exposure and HMGB1 release.** (D)** ATP level of cancer cells treated with Zn-Fu MNs or not measured by ATP Assay Kit. **(E-I)** immune response of mice bearing CT26 tumor treated with PBS, Fu, Zn^2+^, Zn-Fu MNs. **(E)** CD8^+^ T cells and CD4^+^ T cells infiltrate the tumor, measured by flow cytometry. **(F)** The populations of DC cells within spleen, measured by flow cytometry, using CD86 or CD80 as marker. **(G)** The populations of T cells within spleen, measured by flow cytometry, using CD8 or CD4 as marker. **(H)** Interleukin-6 (IL-6) and **(I)** tumor necrosis factor-α (TNF-α) in spleen, which detected by EISA kit. P-values were determined using one-way ANOVA: ***p < 0.001. Error bars denote the standard deviation (n = 3).

Recently, a new immune activation mechanism of ferroptosis inducing cancer cells ICD burst has been found. In the process of ICD, tumor-associated antigens and damage-associated molecular patterns (DAMPs) are exposed from tumor cells, such as cell-surface calreticulin (CRT) 56, high-mobility group box 1 (HMGB1) and ATP (Figure 4A) 57. To study the ability of Zn-Fu MNs induced ICD, those ICD biomarkers were detected after incubating CT26 cancer cells with PBS or Zn-Fu MNs, using appropriate fluorochrome-conjugated anti-mouse antibodies or ATP assay kits 11,12. From confocal images, those cancer cells treated with Zn-Fu MNs displayed the significant increase of CRT exposure in cytoplasm and the great decrease of HMGB1 level within nucleus, in comparison to the control group (Figures 4B, 4C). Moreover, as shown in Figure 4D, we found the dramatic elevation of ATP level was leaked from cancer cells, as the concentration of Zn-Fu MNs increasing. Thus, these results demonstrated that effectively ICD toward CT26 cancer cells could be induced by Zn-Fu MNs via ferroptosis and chemotherapy. These tumor-associated antigens will sensitize naive dendritic cells and augment their antigen presentation capability 11, 58-59. Therefore, we explored the bone marrow derived dendritic cells (BMDCs) maturation after co-incubated with CT26 cells residues *in vitro*. As a result, Zn-Fu MNs treated CT26 residues exhibited more DC cells maturation than control group, further proving the tumor associated antigens release from CT26 cells induced by Zn-Fu MNs (Figure S10).

At present, immune checkpoint blockades (ICB) is one of the main means of tumor immunotherapy, but the depleted T cells in the TME cannot be saved by ICB which resulting in poor treatment effect 60. Therefore, improving the initial activation of T cells and infiltration in tumors is necessary to produce effective antitumor immunity. Among T cells, immune cells called cytotoxic CD8^+^ T cells can directly kill tumor cells, while effector CD4^+^ T cells can activate immune system by sensitizing dendritic cells or other pro-inflammatory myeloid cells. Therefore, enhancing the infiltration of CD8^+^ T cells in tumor and increasing CD4^+^ T cells in immune organs can effectively induce immune response to resist tumors 61. The efficient ICD induction of Zn-Fu MNs encouraged us to explore their immune performances *in vivo*. As shown in the experimental procedure (Figure S11), after treatment tumor with PBS, Fu, Zn^2+^ or Zn-Fu MNs, on day 15, the spleens and tumors were collected from mice for measuring the immune cells via fluorochrome-conjugated antibodies for cell surface markers and the cytokine via enzyme-linked immunosorbent assay (ELISA) kits. From the flow cytometry results showing (Figure 4E), there were enhanced toxic CD8^+^ T cells and assistant CD4^+^ T cells infiltration in tumors, after treatment with Fu or Zn ion, in compassion to control groups. Notably, the tumor treated with Zn-Fu MNs showed the higher T cell infiltration, indicating the higher immune response. From the flow cytometry results of spleen exhibiting (Figure 4F), both Zn^2+^ and Zn-Fu MNs were able to activate more DC cells maturation in spleen, compared with control group. Moreover, Zn-Fu MNs could activate a large number of CD4^+^ T in the spleen for assistant enhanced immunity (Figure 4G). Expectantly, Zn-Fu MNs treated groups displayed the higher secretion level of IL-6 and TNF-α in spleen. Thus, Zn-Fu MNs could induce the higher level of immune response (Figures 4H, 4I).

### *In vivo* antitumor therapy

**Figure 5:**
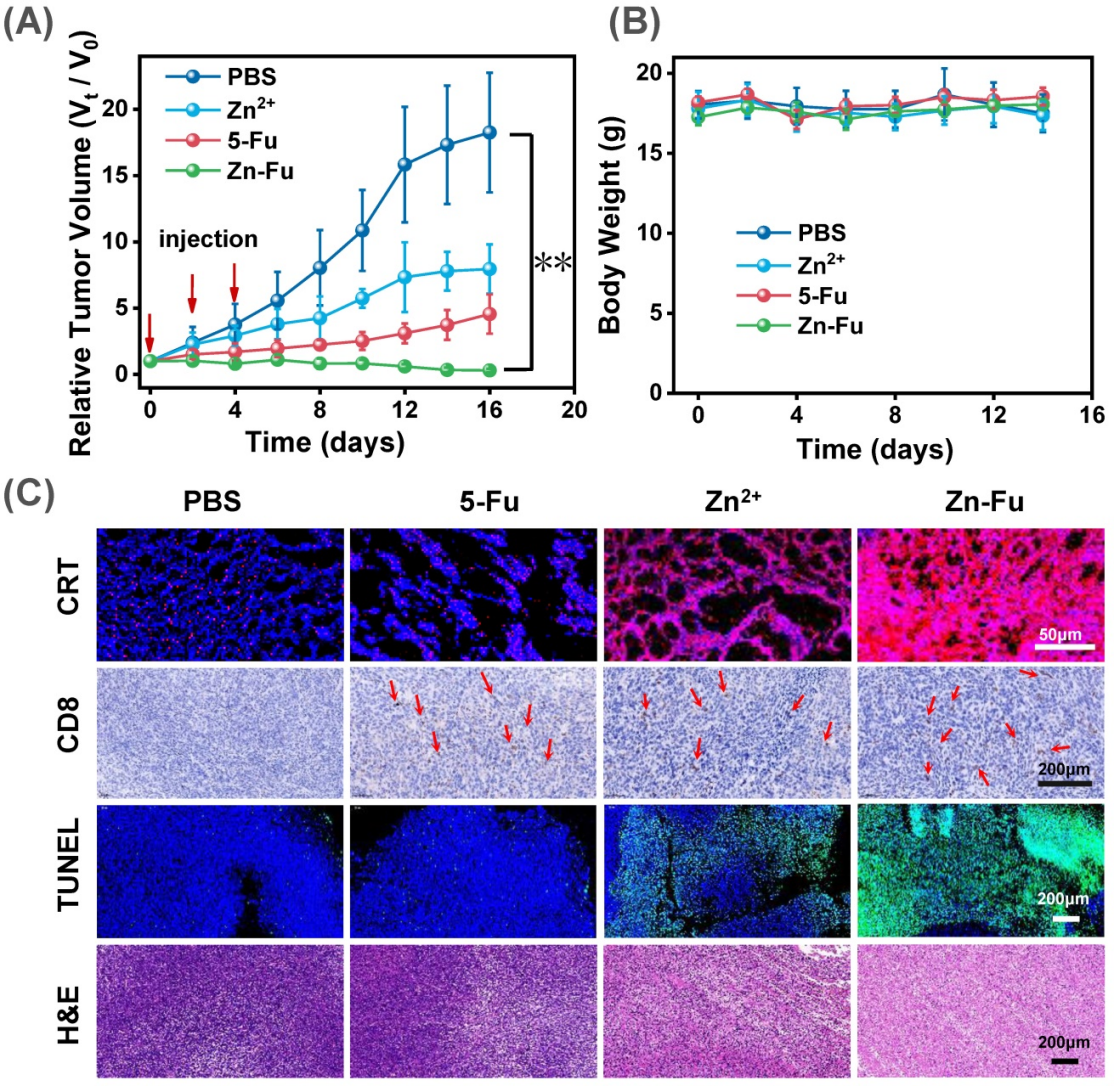
***In vivo***** Antitumor Effect.** The Balb/c mice bore CT26-tumor and received the following treatment: PBS, Fu, Zn^2+^, Zn-Fu MNs on day 0, 2, 4.** (A)** Tumor growth curves and **(B)** body weight of different mice groups. P-values were determined using one-way ANOVA: **p < 0.01. Error bars denote the standard deviation (n = 5).** (C)** CRT, CD8^+^ T, TUNEL, and H&E stain tumor slice for various treatments.

To exam the anticancer outcome of Zn-Fu MNs, as shown in the experimental procedure (Figure S12), CT26-tumor-bearing Balb/c mice received the subcutaneously immunized treatment with injection: PBS, Fu, Zn^2+^, Zn-Fu MNs on day 0, 2, 4. From the tumor growth curves, we found that Fu and Zn^2+^ could partially inhibit the tumor growth, compared with PBS group. Notably, the tumors of Zn-Fu MNs treatment groups were absolutely disappeared, in contrast to other groups (Figure 5A). In addition, those mice were no significant weight loss in all groups during cancer treatment (Figure 5B). Inspired by the efficient ferroptosis induced by Zn-Fu MNs in cancer cells, we explored the GPX4 protein activity and LPO level mediated by Zn-Fu MNs *in vivo*. As shown in Figure S13, Zn-Fu MNs also exhibited good GPX4 down-expression and lipid oxidation within tumor. For pathological examination, the representative tumors were collected from mice on the 10^th^ day post the drug injection. From confocal images of tumor slice, obvious red fluorescence was visualized from Zn-Fu MNs treated group, indicating higher level CRT exposure within tumor. Furthermore, immunohistochemical analysis showed that a large amount of CD8^+^ T cell infiltration within tumor treated by Zn-Fu MNs, compared with PBS group (T cells indicated by arrows). Besides, the representative tumor sections were stained for apoptosis and necrosis analysis using the hematoxylin and eosin (H&E) 62 and terminal deoxynucleotidyl transferase mediated dUTP nick-end labeling (TUNEL) 63. H&E and TUNEL stained tumor slice showed the obvious nuclear condensation and cell contour blurry in H&E observation and increasing green dots in TUNEL images, suggesting severe cell apoptosis and necrosis for tumor treated by Zn-Fu MNs (Figure 5C). Furthermore, we have tested the safety of Zn-Fu MNs in blood. Compared with control group, Zn-Fu MNs treated mice showed no significant changes of main indexes for blood biochemistry and complete blood panel analysis, which confirmed the safety of Zn-Fu MNs* in vivo* (Figure S14). Moreover, main organs from Zn-Fu MNs, exhibited no noticeable pathological damages after stained tissue slices with H&E (Figure S15). These results demonstrated the low side toxicity of Zn-Fu MNs for immunological therapy.

## Discussion

Recently, the ferroptosis has been considered as a promising method in reinforcing immune responses of anti-tumor through inducing ICD of cancer cells 11. Most of ferroptosis agents reported were iron-based nanomaterials such as Fe_3_O_4_ or single-crystal Fe (0) that produce oxidative stress in cancer cells based on Fenton reaction by catalyzing endogenous H_2_O_2_ 18,19. However, the tumor microenvironment of slightly acidic (pH 5.5-6.5) resulted in the low ROS generation efficacy of Fe^2+^. Encouragingly, other metal ions have been developed to enhance Fenton reaction efficacy and ROS production 11,17. However, the insufficient intracellular H_2_O_2_ still limits the accumulation of oxidation products, leading to inadequate ferroptosis and unsatisfactory immune responses. Thus, it is still a great challenge to seek an effective method to develop ferroptosis medicine which can effectively activate immunity.

Compared with other ferroptosis strategies for improving the ROS levels within tumor such as Fenton reaction or photodynamic effect, our strategy developed to inactivate mitochondrial electron transport thereby enhance ROS production efficacy. Specifically, in our study, we prepared Zn-Fu MNs by simply coordinating fluorouracil and zinc nitrate. Due to the facile one-pot preparation, Zn-Fu MNs exhibited the high-drug loading efficiency (40.93%), and high production efficiency (160 mg/pot). Moreover, Zn-Fu MNs were a double stimulus activated nanoplatform responding to the tumor microenvironment (low acidity and high concentration of ATP), owing to the easy protonation of nitrogen atom and the Zn coordination competition between ATP and Fu 36,41. Thus, Zn-Fu MNs were stable in normal tissue microenvironment (pH 7.4 and low concentration of ATP), but completely decomposed within tumor (Figures 2C-2F) 40. Moreover, this dual-responsive property of Zn-Fu MNs was able to reduce the premature release of drugs at physiological pH levels, and promote more effective drug release within tumor cells.

Furthermore, we found the correlation between the simultaneous release of Fu and the variation of ^19^F-MRI signals. As expected, no obvious ^19^F NMR signal was detected at pH 7.4, indicating the ^19^F signals of Zn-Fu MNs was completely “quenched”, due to the dense structure of Zn-Fu nanoplatform that was able to restrict the mobility of ^19^F atoms 32. Interestingly, with the decrease of pH, the ^19^F MRI signal was gradually recovered (Figure 2G). As expected, we found that ATP can also amplify the ^19^F MR signal, which was attributed to the acidity + ATP dual triggered release of Fu molecules (Figure 2H). Thereby, we have successfully achieved a good relevance between the release of Fu and ^19^F MR imaging signals.

During cancer therapeutic process, we found that zinc ions released from Zn-Fu MNs was able to damage mitochondria, the main site of ROS production, to generate severe inflammatory reaction (Figures 3B, 3C) 47 which induced GSH depletion and GPX4 down-expression (Figures 3E, 3F). The ROS generation and GPX4 inactivation led to lipid oxidation (Figure 3G) and MDA accumulation in cancer cells (Figure 3H). Meanwhile, the release of Fu resulted in the abnormal transcription by participating in DNA replication in cancer cells (Figure 3I). Therefore, the combination of Fu-mediated apoptosis by DNA damages with Zn-mediated ferroptosis by mitochondrial dysfunction endowed Zn-Fu MNs with greatly increasing anti-cancer effects in tumor therapy (Figure 3J).

Next, we explored the immune response efficiency of Zn-Fu MNs. Under the synergistic effect of Zn and 5-Fu, those tumor cells, treated with Zn-Fu MNs, released various antigens such as CRT, HMGB1 and ATP, which would enhance the activation of immune cells. Finally, the potential immune performances of Fu, Zn^2+^ and Zn-Fu MNs had been studied *in vivo* anti-tumor therapy (Figures 4E-4I). We found that Fu alone could induce a large number of T cells infiltration into tumor, but its immune enhancement effect was not very well, probably because the toxicity of DNA replication disorder induced by Fu was not strong enough to activate systemic immunity 24. As a complement, zinc was able to induce tumor ferroptosis, which could activate the maturation of a large number of immune cells in the spleen. As expected, Zn-Fu MNs enabled to activate the maturation of large number of immune cells in the spleen and promote the infiltration toxic of T cells within tumor. Compared with traditional immune inducers, Zn-Fu MNs was able to cause a strong immune response without loading redundant antigens, showing an excellent therapeutic effect in CT26-bearing mice with little side toxicity (Figures 5A-5C).

## Conclusion

A novel Zn-Fu metallodrug networks as immune inducer was synthesized by a one-pot method with high-drug loading efficiency (40.93 %). Within tumor microenvironment, the weak acidity and high level of ATP could trigger the decomposition of Zn-Fu MNs with^19^F-MRI signal to be switched on for correlating with drug release. Importantly, the release of Zn^2+^ ions was able to stimulate mitochondria to violently produce reactive oxygen species and thereby induce ferroptosis, while the release of Fu could inhibit cancer cells growth via interfering with DNA synthesis of cells. Moreover, Zn-Fu MNs enabled to serve as a potent ICD drug, due to the combination of Fu-mediated chemotherapy and Zn^2+^- mediated ferroptosis. As a result, Zn-Fu MNs as immune inducers achieved the maturation of immune cells in spleen, as well as the infiltration of CD8^+^ T cells within tumor, resulting in the significant inhibition of cancer growth for tumor bearing mice.

## Materials and methods

### Synthesis of Zn-Fu MNs

300 mg Zn (NO_3_)_2_·6H_2_O (1 mmoL) and 130 mg 5-Fu (1 mmoL) was evenly dissolved in 30 mL N, N - Dimethylformamide (DMF) under ultrasound. Then, 250 μL triethylamine was added to the mixture solution and sonicated for 60 min. After that, the mixture solution was transferred to a baking oven and heat to 100 °C for 60 min. After heating reaction, the sample was cooling down to room temperature and sonicated kept for 20 min. The obtained sample was washed with DMF and ultrapure water for three times, respectively 36. The drug-loading efficacy was assessment via the following calculation formulas:

Drug-loading efficiency (%) = 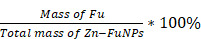


### The release of Fu and Zn^2+^ *in vitro*

Zn-Fu MNs (0.4 mg/mL) were incubated with 1×Tris buffer at pH 5.4 or 7.4. At different time points, the supernatant fluid was obtained by centrifugation and then fresh PBS buffer at pH 5.4 or 7.4 was added. The supernatant fluid was measured for Fu using UV-Vis absorbance spectrometer and Zn^2+^ concentrations using ICP-MS.

### For double response release of acid and ATP

Zn-Fu MNs (0.4 mg/mL) were incubated with different condition with 1×Tris buffer: (a) pH 7.4 + 0.4 mM ATP; (b) pH 7.4 + 5 mM ATP; (c) pH 5.4 + 5 mM ATP. The release of Fu and Zn^2+^ were detected as the methods mentioned above.

### pH-responsive ^19^F NMR and MR imaging (^19^F-MRI) *in vitro*

For accurate quantification of ^19^F NMR signals, Zn-Fu MNs (4 mg/mL) were incubated with different condition with 1×PBS buffer: (a) pH 7.4; (b) pH 6.4; (c) pH 5.4; (d) pH 4.4; (e) pH 3.4, containing 10% D_2_O for shimming and sodium trifluoroacetate (F = 3.5 mM) as internal standard. Spectra were acquired were obtained by Avance III HD 300 MHz NMR spectrometer (Bruker). In addition, ^19^F MR imagine of all samples were collected from 7.0 T MRI scanner. The ^19^F MRI acquiring parameters were as follows: size = 256 × 256, field of view [FOV] = 3.0 cm × 3.0 cm, slice thickness = 10 mm, repetition time [TR] = 2.4 ms, effective echo time [TE] = 10 ms, average = 1000 times, frequency = -82.5 ppm, and the reference power = 0.33 W).

### ATP and pH-responsive ^19^F NMR and MR imaging (^19^F-MRI) *in vitro*

The sodium trifluoroacetate (F = 3.5 mM) and Zn-Fu MNs (4 mg/mL) were incubated with (a) pH 7.4; (b) pH 7.4 + 0.4 mM ATP; (c) pH 7.4 + 10 mM ATP; (d) pH 6.4; (e) pH 6.4 + 0.4 mM ATP; (f) pH 5.4; (g) pH 6.4 + 10 mM ATP. At the same method, the ^19^F NMR of Zn-Fu MNs were obtained. In addition, to compare the effects of different conditions on ^19^F NMR of Zn-Fu MNs, we selected the CF_3_COONa as the internal reference. By adding the same concentration of CF_3_COONa, the signal intensity of Zn-Fu MNs was compared under the same internal reference intensity.

### Cellular experiments

CT26 cells were selected for cell experiment verification. CT26 cells were obtained from ATCC (USA), and aseptically cultured in standard 1640 culture media with 10% fetal bovine serum and 1% penicillin/streptomycin (37 °C, 5% CO_2_).

### For intracellular reactive oxygen species (ROS) detection

For *in vitro* ROS detection, the CT26 cells were cultured in confocal glass dish (2 × 10^6^ cells/dish) and incubated with (a) PBS; (b) 5-Fu (20 μg/mL); (c) Zn (NO_3_)_2_ (Zn^2+^ 10 μg/mL); (d) Zn-Fu MNs (50 μg/mL, contain 5-Fu 20 μg/mL and Zn^2+^ 10 μg/mL). After 12 h dark incubation, samples washed by PBS for three times and then re-incubated in fresh culture medium with 2',7'-dichlorodihydrofluorescein (DCFH-DA) kept for 30 min at 37 °C. Finally, the fluorescent intensity of 2',7'-dichlorofluorescein (DCF, Ex = 488 nm) was imagined using laser scanning confocal microscopy (LSCM). For quantitative analysis of DCF signal, the single cell suspension was obtained using trypsinization and analyzed by flow cytometry.

### For lysosomal membrane integrity detection

CT26 cells after incubation with Zn-Fu MNs were evaluated by the acridine orange (AO) staining method. Briefly, CT26 cells were seeded in confocal glass dish with 0.8 × 10^5^ cells per dish and subjected to treated with (a) PBS; (b) 5-Fu (20 μg/mL); (c) Zn (NO_3_)_2_ (Zn^2+^ 10 μg/mL); (d) Zn-Fu MNs (50 μg/mL, contain 5-Fu 20 μg/mL and Zn^2+^ 10 μg/mL). After 12 h of dark incubation, PBS washed samples for three times and re incubated in fresh culture medium. Then, each well was added with the AO probe (10 μM) and kept for 30 min at 37 °C. Finally, the fluorescence (Ex = 488 or 561 nm) was detected by LSCM.

### Mitochondrial membrane potential detection

The detection of mitochondrial membrane potential used JC-1 probe. Briefly, CT26 were seeded in confocal glass culture dish with 0.8 × 10^5^ cells per dish. After 12 h treatment with (a) PBS; (b) 5-Fu (20 μg/mL); (c) Zn (NO_3_)_2_ (Zn^2+^ 10 μg/mL); (d) Zn-Fu MNs (50 μg/mL contain 5-Fu 20 μg/mL and Zn^2+^ 10 μg/mL), cells were washed with PBS and the JC-1 (10 μg/mL) probe was added for 30 min. Then cells were rinsed three times by PBS and detected by LSCM. JC-1 monomers adopt green channel with 488 nm excitation wavelength, while JC-1 aggregates adopt red channel with 546 nm excitation wavelength.

### GSH consumption detection

CT26 cells were cultured in 6-well dish with a density of 2 × 10^6^ cells per well and co-incubated with (a) PBS; (b) 5-Fu (20 μg/mL); (c) Zn (NO_3_)_2_ (Zn^2+^ 10 μg/mL); (d) Zn-Fu MNs (50 μg/mL, contain 5-Fu 20 μg/mL and Zn^2+^ 10 μg/mL), respectively. After 24 h of dark incubation, the cells were washed with PBS and collected for GSH detection by the GSH assay kit according to the manufacturer's protocols (Ex = 412 nm).

### Western blot

For western blots of GPX4 expressions, CT26 cells were seeded in culture dish with 3 × 10^6^ cells per dish and co-incubated with (a) PBS; (b) 5-Fu (20 μg/mL); (c) Zn(NO_3_)_2_ (Zn^2+^ 10 μg/mL); (d) Zn-Fu MNs (50 μg/mL, 100 μg/mL, 150 μg/mL, 200 μg/mL) for 24 h, respectively. Next, those cells were disrupted, and the cell lysate was collected and boiled for 10 min at 95 °C. Subsequently, the cell lysates underwent SDS-polyacrylamide gel electrophoresis operation and were further transferred to PVDF membrane. Then, the membrane was blocked with tris buffered with Tween-20 + 5% skim milk, and immunoblotted with GAPDH antibody (1: 2000, Servicebio) and rabbit GPX4 antibody (1: 1000, Absin) for overnight (4 °C), followed by the further incubation of secondary antibody labeled horseradish peroxidase (HRP) (1: 10000, YiShan Biotech) for 1 h (37 °C). Finally, the specific protein bands were captured with an enhanced chemiluminescent detection system.

### Lipid peroxidation (LPO) detection

LPO was further examined by a LPO probe Liperfluo. Briefly, CT26 cells were cultured in culture dish with 2 × 10^5^ cells per dish. First, CT26 cells were treated with (a) PBS; (b) 5-Fu (20 μg/mL); (c) Zn(NO_3_)_2_ (Zn^2+^ μg/mL); (d) Zn-Fu MNs (50 μg/mL, contain 5-Fu 20 μg/mL and Zn^2+^ 10 μg/mL) solution diluted with serum-free 1640 medium for 24 h, then incubated with Liperfluo probe (5 μM) for 30 min at 37 °C. Finally, after washed with PBS, the fluorescence was further measured by flow cytometry (LPO: Ex = 488 nm, Em = 530 nm).

### MDA detection

The extracellular produced MDA were examined using the Lipid oxidation (MDA) detection kit. CT26 cells were seeded in 6-well culture dish with 3 × 10^6^ cells per well and co-incubated with (a) PBS; (b) 5-Fu (20 μg/mL); (c) Zn (NO_3_)_2_ (Zn^2+^ 10 μg/mL); (d) Zn-Fu MNs (50 μg/mL, contain 5-Fu 20 μg/mL and Zn^2+^ 10 μg/mL), respectively. After 24 h of dark incubation, the cells were washed with cold PBS and collected for MDA detection by the Lipid oxidation (MDA) detection kit and the method refers to the manufacturer's protocols.

### DNA damage evaluation

Here, CT26 cells were cultured in confocal glass dish with 1 × 10^5^ cells per dish and incubated with (a) PBS; (b) 5-Fu (20 μg/mL); (c) Zn (NO_3_)_2_ (Zn^2+^ 10 μg/mL); (d) Zn-Fu MNs (50 μg/mL, contain 5-Fu 20 μg/mL and Zn^2+^ 10 μg/mL) for 6 h in cell incubator. Next, the anti-phospho-histone γ-H_2_AX mouse monoclonal antibody (dilution 1:1000) was added for 24 h at 4 °C. Then the cells were washed with cold PBS and incubated with sheep anti-mouse secondary antibody labeled Cy3 (dilution 1:2000) for another 2 h at 37 °C. Finally, cells were stained with DAPI for nuclear localization about 15 min and imaged by LSCM.

### For *in vitro* therapy

CT26 cells were placed in a 96-well plate with 2×10^3^ cells per well. After 24 h incubation, the culture medium was replaced by fresh 1640 with different concentrations of Zn-Fu MNs for another 24 h at 37 °C. Then, each well cells were pre-cultured in 100 μL of free 1640 culture medium with 10 μL MTT reagent for 4 h at 37 °C. The relative cell viability was detected according to the standard methyl thiazolyl tetrazolium (MTT) method (Ex = 490 nm) (Bio-Tek, Winooski, VT).

### Calreticulin expression and high-mobility group box 1 released detections

To detect CRT expression in cytoplasm, briefly, CT26 cells were seeded in confocal glass dish with 1 × 10^5^ cells per well and co-incubated with (a) PBS; (b) Zn-Fu MNs (50 μg/mL). After 24 h treatment in dark, the cells fixed by paraformaldehyde and then stained with anti-calreticulin antibody for 12 h at 4 °C. Subsequently, the cells were washed with cold PBS and incubated with secondary antibody labeled Cy5 for 1 h (37 °C). Then the cell nuclear were stained with DAPI for 15 min and detected under LSCM. The high-mobility group box 1 (HMGB1) released was detected using same method (DAPI: Ex = 405 nm; CRT: Ex = 637 nm; HMGB1: Ex = 561 nm).

### Adenosine triphosphate (ATP) detections

The extracellular released ATP were examined using the ATP assay Kit. Briefly, CT26 cells were cultured in 6-well culture dish (3 × 10^5^ cells/well) and then co-incubated with PBS or Zn-Fu MNs (50 μg/mL, 100 μg/mL). After 12 h of dark incubation, the culture media was collected via a centrifugation at 4 °C (2500 rpm, 4 min). The supernatant content of ATP was measured by the ATP assay Kit and the method refers to the manufacturer's protocols.

### Animal experiment

All animal experiments were complied with Animal Care and Use Committee of Hunan University. Female Balb/c mice were purchased from Hunan SJA Laboratory Animal Co., Ltd. and fed in an environmentally manageable animal laboratory. In order to establish the tumor model, 50 µL CT26 cells solution (1×10^6^ cells) were inoculated into the true subcutaneous back area of mice.

### Quantification of cytokines, dendritic cells and T cells in spleen

Here, to evaluate the potential immune performances of Zn-Fu MNs, the experimental process is shown in supplementary figure S11. CT26 tumor-bearing Balb/c mice were randomly divided into 4 groups (n = 3), which were subcutaneously immunized (near tumors) with injections of (1) PBS; (2) Zn(NO_3_)_2_ (50 µg Zn^2+^ / mouse, 20 µL); (3) 5-Fu (100 µg 5-Fu/mouse, 20 µL); (4) Zn-Fu MNs (250 µg Zn-Fu/mouse, 20 µL, contain Zn^2+^ 50 µg and 5-Fu 100 µg) on day 7, 9, 11, respectively. The mice were sacrificed and their spleens and tumors were collected on day 15, then the spleen was mashed and passed through 70 µm cell strainer supported by 50 mL of polypropylene pipe using 2 mL of 1 × PBS buffer as lubricants. The IL-6 and TNF-α ELISA kits were used to detect cytokines in the supernatant samples from minced spleens. The isolated cells of spleen were stained with appropriate fluorochrome-conjugated anti-mouse antibodies: (1) anti-mouse CD3, anti-mouse CD4 APC, anti-mouse CD8 PE; (2) anti-CD11c FITC, anti-CD80 PE and anti-CD86 APC (Biolegend), for cell surface markers and analyzed by flow cytometry. For the infiltration of T cells, the isolated cells of tumors were obtained by shearing, collagenase digestion and trypsinization. The reaction was stopped by 1640 complete medium. Then the cells were washed twice in PBS and stained with appropriate fluorochrome-conjugated anti-mouse antibodies: anti-mouse CD3 FITC, anti-mouse CD4 APC, anti-mouse CD8 PE, for cell surface markers and analyzed by flow cytometry.

### *In vivo* anti-tumor effect in a CT26 tumor model

CT26 cells were inoculated into the right back of Balb/c mice (1.5 × 10^6^ cells per mouse).And after seven days, these CT26 tumor-bearing Balb/c mice were randomly divided into 4 groups (n = 5), which were subcutaneously immunized (near primary tumors) with injections of (1) PBS; (2) Zn(NO_3_)_2_ (50 µg Zn^2+^ / mouse, 20 µL); (3) 5-Fu (100 µg 5-Fu / mouse, 20 µL); (4) Zn-Fu MNs (250 µg Zn-Fu / mouse, 20 µL, contain Zn^2+^ 50 µg and 5-Fu 100 µg) every 2 days for three times. From the time of treatment, the body weight and tumor size of mice were measured every 2 days. The tumor volume was calculated through length ×width^2^ / 2 of tumor. The tumor growth rate was calculated by dividing the tumor volume measured at each time by the initial tumor volume before the first inoculation. After 17^th^ day post the first injection, the representative mice were selected and their organs (heart, liver, kidney, lung and spleen) were sectioned for H&E staining after sacrificed.

### Histological analysis

Tumor sections were stained with fluorochrome-conjugated anti-mouse CD8 antibodies and CRT antibodies, haematoxylin and eosin (H&E) and terminal deoxynucleotidyl transferase-mediated dUTP nick-end labeling (TUNEL), then observed under fluorescence microscope. Briefly, the CT26 tumor-bearing Balb/c mice with an average tumor volume of 150 mm^3^ were randomly allocated into 4 groups (n = 3), which were subcutaneously immunized (near tumors) with (1) PBS; (2) Zn(NO_3_)_2_ (25 µg Zn^2+^/mouse, 20 µL); (3) 5-Fu (50 µg 5-Fu/mouse, 20 µL); (4) Zn-Fu MNs (125 µg Zn-Fu/mouse, 20 µL, contain Zn^2+^ 25 µg and 5-Fu 50 µg) every 2 days for three times. At the 10^th^ day post first-treatment, all of mice were sacrificed, their tumors were collected and sliced:

For evaluating CRT exposure within tumor: those tumor slices were stained with anti-calreticulin primary antibody at 37 °C for 2 h, with APC-conjugated secondary antibody at 37 °C for 30 min, and with DAPI for 15 min, successively. Finally, the tumor slices were observed by the confocal laser scanning microscope (DAPI: Ex = 405 nm; CRT: Ex = 637 nm).

In order to assess the enrichment of CD8^+^ T cells: The tumor tissue sections were stained with CD8 antibody and observed the slices with a Pannoramic MIDI microscope (3DHIESTECH, Hungary).

For TUNEL staining, the tumor slices sectioned into 4 μm slices using a conventional microtome, stained with TUNEL and DAPI, and examined using a Pannoramic MIDI microscope (DAPI: Ex = 405 nm; TUNEL: Ex = 488 nm).

For tumor H&E staining, the process staining was similar with that of TUNEL staining.

## Supplementary Material

Supplementary methods and figures.

## References

[1] Wang T, Wang D, Yu H, Feng B, Zhou F, Zhang H (2018). A cancer vaccine-mediated postoperative immunotherapy for recurrent and metastatic tumors. Nat Commun.

[2] Harjes U (2017). Tumour immunology: Tumours copy to escape. Nat Rev Cancer.

[3] Nunes JB, Everts B (2019). Metabolic stress triggers immune escape by tumors. Trends Cancer.

[4] Schreiber H, Wu TH, Nachman J, Kast WM (2002). Immunodominance and tumor escape. Semin Cancer Biol.

[5] Dai Z, Tang J, Gu Z, Wang Y, Yang Y, Yang Y (2020). Eliciting immunogenic cell death via a unitized nanoinducer. Nano Lett.

[6] Jiang W, Wang L, Wang Q, Zhou H, Ma YC, Dong W (2021). Reversing immunosuppression in hypoxic and immune-cold tumors with ultrathin oxygen self-supplementing polymer nanosheets under near infrared light irradiation. Adv Funct Mater.

[7] Zheng P, Ding B, Jiang Z, Xu W, Li G, Ding J (2021). Ultrasound-augmented mitochondrial calcium ion overload by calcium nanomodulator to induce immunogenic cell death. Nano Lett.

[8] Li J, Zhou S, Yu J, Cai W, Yang Y, Kuang X (2021). Low dose shikonin and anthracyclines coloaded liposomes induce robust immunogenetic cell death for synergistic chemo-immunotherapy. J Control Release.

[9] Au KM, Balhorn R, Balhorn MC, Park SI, Wang AZ (2019). High-performance concurrent chemo-immuno-radiotherapy for the treatment of hematologic cancer through selective high-affinity ligand antibody mimic-functionalized doxorubicin-encapsulated nanoparticles. ACS Cent Sci.

[10] Chen Q, Chen M, Liu Z (2019). Local biomaterials-assisted cancer immunotherapy to trigger systemic antitumor responses. Chem Soc Rev.

[11] Ding B, Zheng P, Jiang F, Zhao Y, Wang M, Chang M (2020). MnO_x_ nanospikes as nanoadjuvants and immunogenic cell death drugs with enhanced antitumor immunity and antimetastatic effect. Angew Chem Int Ed.

[12] Ding B, Shao S, Yu C, Teng B, Wang M, Cheng Z (2018). Large-pore mesoporous-silica-coated upconversion nanoparticles as multifunctional immunoadjuvants with ultrahigh photosensitizer and antigen loading efficiency for improved cancer photodynamic immunotherapy. Adv Mater.

[13] Zhao LP, Zheng RR, Huang JQ, Chen XY, Deng FA, Liu YB (2020). Self-delivery photo-immune stimulators for photodynamic sensitized tumor immunotherapy. ACS Nano.

[14] Pan J, Hu P, Guo Y, Hao J, Ni D, Xu Y (2020). Combined magnetic hyperthermia and immune therapy for primary and metastatic tumor treatments. ACS Nano.

[15] Yang WS, SriRamaratnam R, Welsch ME, Shimada K, Skouta R, Viswanathan VS (2014). Regulation of ferroptotic cancer cell death by GPX4. Cell.

[16] Xu G, Wang H, Li X, Huang R, Luo L (2021). Recent progress on targeting ferroptosis for cancer therapy. Biochem Pharmacol.

[17] Du Y, Zhang R, Yang J, Liu S, Zhou J, Zhao R (2022). A “closed-loop” therapeutic strategy based on mutually reinforced ferroptosis and immunotherapy. Adv Funct Mater.

[18] Song R, Li T, Ye J, Sun F, Hou B, Saeed M (2021). Acidity-activatable dynamic nanoparticles boosting ferroptotic cell death for immunotherapy of cancer. Adv Mater.

[19] Liang H, Wu X, Zhao G, Feng K, Ni K, Sun X (2021). Renal clearable ultrasmall single-crystal Fe nanoparticles for highly selective and effective ferroptosis therapy and immunotherapy. J Am Chem Soc.

[20] Chen Q, Liu L, Lu Y, Chen X, Zhang Y, Zhou W (2019). Tumor microenvironment-triggered aggregated magnetic nanoparticles for reinforced image-guided immunogenic chemotherapy. Adv Sci.

[21] Liang C, Zhang X, Yang M, Dong X (2019). Recent progress in ferroptosis inducers for cancer therapy. Adv Mater.

[22] De Angelis PM, Svendsrud DH, Kravik KL, Stokke T (2006). Cellular response to 5-fluorouracil (5-Fu) in 5-Fu-resistant colon cancer cell lines during treatment and recovery. Mol Cancer.

[23] Ghiringhelli F, Apetoh L (2015). Enhancing the anticancer effects of 5-fluorouracil: Current challenges and future perspectives. Biomed J.

[24] Gmeiner WH (2020). Fluoropyrimidine modulation of the anti-tumor immune response-prospects for improved colorectal cancer treatment. Cancers.

[25] Longley DB, Harkin DP, Johnston PG (2003). 5-fluorouracil: Mechanisms of action and clinical strategies. Nat Rev Cancer.

[26] Xu H, Yang T, Liu X, Tian Y, Chen X, Yuan R (2016). Luteolin synergizes the antitumor effects of 5-fluorouracil against human hepatocellular carcinoma cells through apoptosis induction and metabolism. Life Sci.

[27] Fan XJ, Jiao GZ, Zhao W, Jin PF, Li X (2013). Magnetic Fe_3_O_4_-graphene composites as targeted drug nanocarriers for pH-activated release. Nanoscale.

[28] Mitra S, Sasmal HS, Kundu T, Kandambeth S, Illath K, Díaz Díaz D (2017). Targeted drug delivery in covalent organic nanosheets (CONs) via sequential postsynthetic modification. J Am Chem Soc.

[29] Liu W, Li XL, Wong Y-S, Zheng WJ, Zhang YB, Cao WQ (2012). Selenium nanoparticles as a carrier of 5-fluorouracil to achieve anticancer synergism. ACS Nano.

[30] Agasti SS, Chompoosor A, You C-C, Ghosh P, Kim CK, Rotello VM (2009). Photoregulated release of caged anticancer drugs from gold nanoparticles. J Am Chem Soc.

[31] Zhang C, Moonshi SS, Wang W, Ta HT, Han Y, Han FY (2018). High F-content perfluoropolyether-based nanoparticles fortargeted detection of breast cancer by (19)F magnetic resonance and optical imaging. ACS Nano.

[32] Tang X, Gong X, Li A, Lin H, Peng C, Zhang X (2020). Cascaded multiresponsive self-assembled (19)F MRI nanoprobes with redox-triggered activation and NIR-induced amplification. Nano Lett.

[33] Jahromi AH, Wang C, Adams SR, Zhu W, Narsinh K, Xu H (2019). Fluorous-soluble metal chelate for sensitive fluorine-19 magnetic resonance imaging nanoemulsion probes. ACS Nano.

[34] Brix G, Bellemann ME, Haberkorn U, Gerlach L, Lorenz WJ (1996). Assessment of the biodistribution and metabolism of 5fluorouracil as monitored by ^18^F PET and ^19^F MRI: A comparative animal study. Nucl Med Biol.

[35] Brix G, Bellemann ME, Gerlach L, Haberkorn U (1999). Direct detection of intratumoral 5-fluorouracil trapping using metabolic ^19^F MR imaging. Magn Reson Imaging.

[36] Yang C, Song GS, Yuan HF, Yang Y, Wang YQ, Ye DJ (2020). Manganese- fluorouracil metallodrug nanotheranostic for MRI correlated drug release and enhanced chemoradiotherapy. CCS Chem.

[37] Tan MX, Chen YL, Guo Y, Yang C, Liu MZ, Guo D (2020). A low-intensity focused ultrasound-assisted nanocomposite for advanced triple cancer therapy: local chemotherapy, therapeutic extracellular vesicles and combined immunotherapy. Biomater Sci.

[38] Tan MX, Liu WW, Liu FQ, Zhang W, Gao H, Cheng J (2019). Silk fibroin-coated nanoagents for acidic lysosome targeting by a functional preservation strategy in cancer chemotherapy. Theranostics.

[39] Yang XT, Tang Q, Jiang Y, Zhang MN, Wang M, Mao LQ (2019). Nanoscale ATP-responsive zeolitic imidazole framework-90 as a general platform for cytosolic protein delivery and genome editing. J Am Chem Soc.

[40] Mo R, Jiang TY, DiSanto R, Tai WY, Gu Z (2014). ATP-triggered anticancer drug delivery. Nat Commun.

[41] Deng J, Wang K, Wang M, Yu P, Mao L (2017). Mitochondria targeted nanoscale zeolitic imidazole framework-90 for ATP imaging in live cells. J Am Chem Soc.

[42] Lin H, Tang X, Li A, Gao J (2021). Activatable (19)F MRI nanoprobes for visualization of biological targets in living subjects. Adv Mater.

[43] Lin LS, Wang JF, Song JB, Liu YJ, Zhu GZ, Dai YL (2019). Cooperation of endogenous and exogenous reactive oxygen species induced by zinc peroxide nanoparticles to enhance oxidative stress-based cancer therapy. Theranostics.

[44] Zhang C, Liu Z, Zhang Y, Ma L, Song E, Song Y (2020). "Iron free" zinc oxide nanoparticles with ion-leaking properties disrupt intracellular ros and iron homeostasis to induce ferroptosis. Cell Death Dis.

[45] Palmer LD, Jordan AT, Maloney KN, Farrow MA, Gutierrez DB, Gant-Branum R (2019). Zinc intoxication induces ferroptosis in A549 human lung cells. Metallomics.

[46] Dai Z, Wang Q, Tang J, Wu M, Li H, Yang Y (2022). Immune-regulating bimetallic metal-organic framework nanoparticles designed for cancer immunotherapy. Biomaterials.

[47] Dong SM, Dong YS, Liu B, Liu J, Liu SK, Zhao ZY (2022). Guiding transition metal-doped hollow cerium tandem nanozymes with elaborately eegulated multi-enzymatic activities for intensive chemodynamic therapy. Adv Mater.

[48] Dong YS, Dong SM, Liu B, Yu CH, Liu J, Yang D (2021). 2D piezoelectric Bi_2_MoO_6_ nanoribbons for GSH-enhanced sonodynamic therapy. Adv Mater.

[49] Liu Y, Zhai S, Jiang X, Liu Y, Wang K, Wang C (2021). Intracellular mutual promotion of redox homeostasis regulation and iron metabolism disruption for enduring chemodynamic therapy. Adv Funct Mater.

[50] Yuan H, Han Z, Chen Yc, Qi F, Fang HB, Guo ZJ (2021). Ferroptosis photoinduced by new cyclometalated iridium(iii) complexes and its synergism with apoptosis in tumor cell inhibition. Angew Chem Int Ed.

[51] Li Y, Zhang R, Wan Q, Hu R, Ma Y, Wang Z (2021). Trojan horse-like nano-AIE aggregates based on homologous targeting strategy and their photodynamic therapy in anticancer application. Adv Sci.

[52] Neale TJ, Ojha PP, Exner M, Poczewski H, Ruger B, Witztum JL (1994). Proteinuria in passive heymann nephritis is associated with lipid peroxidation and formation of adducts on type iv collagen. J Clin Invest.

[53] Zhao Y, Kong W, Wang P, Song G, Song ZL, Yang Y (2021). Tumor-specific multipath nucleic acid damages strategy by symbiosed nanozyme@enzyme with synergistic self-cyclic catalysis. Small.

[54] Shi LN, Wang YG, Zhang C, Zhao Y, Lu C, Yin BL (2021). An acidity-unlocked magnetic nanoplatform enables self-boosting ROS generation through upregulation of lactate for imaging-guided highly specific chemodynamic therapy. Angew Chem Int Ed.

[55] Zhang HL, Hu BX, Li ZL, Du T, Shan JL, Ye ZP (2022). PKCβII phosphorylates ACSL4 to amplify lipid peroxidation to induce ferroptosis. Nat Cell Biol.

[56] Obeid M, Tesniere A, Ghiringhelli F, Fimia GM, Apetoh L, Perfettini JL Calreticulin exposure dictates the immunogenicity of cancer cell death. Nat Med. 12007; 13: 54-61.

[57] Wong DYQ, Ong WWF, Ang WH, Induction of Immunogenic Cell Death by Chemotherapeutic Platinum Complexes Angew Chem Int Ed. 2015; 54: 6483-7.

[58] Lin H, Yang C, Luo Y, Ge M, Shen H, Zhang XL, Shi JL (2022). Biomimetic nanomedicine-triggered *in situ* vaccination for innate and adaptive immunity activations for bacterial osteomyelitis treatment. ACS Nano.

[59] Chen Y, Wang M, Zheng K, Ren YG, Xu H, Yu ZZ Antimony nanopolyhedrons with tunable localized surface plasmon resonances for highly effective photoacoustic imaging-guided synergistic photothermal/immunotherapy Adv Mater. 2021; 33: 2100039.

[60] Feng B, Zhou F, Hou B, Wang D, Wang T, Fu Y (2018). Binary cooperative prodrug nanoparticles improve immunotherapy by synergistically modulating immune tumor microenvironment. Adv Mater.

[61] Binnewies M, Mujal AM, Pollack JL, Combes AJ, Hardison EA, Barry KC (2019). Unleashing type-2 dendritic cells to drive protective antitumor CD4(+) T cell immunity. Cell.

[62] Cheng W, Cheng H, Wan S, Zhang X, Yin M (2017). Dual-stimulus-responsive fluorescent supramolecular prodrug for antitumor drug delivery. Chem Mater.

[63] Wang YJ, Song GS, Liao SY, Qin QQ, Zhao Y, Shi LN (2021). Cyclic amplification of the afterglow luminescent nanoreporter enables the prediction of anti-cancer efficiency. Angew Chem Int Ed.

