# Supplementary material for: Metal-fluorouracil networks with disruption of mitochondrion enhanced ferroptosis for synergistic immune activation
Source: Theranostics. 2022 Aug 21;12(14):6207–22. doi: 10.7150/thno.75323 (PMC9475458; doi:10.7150/thno.75323)
Supplement: Supplementary file 1 — Supplementary methods and figures. [file thnov12p6207s1.pdf]

Supplementary Information (SI)

# Metal-fluorouracil networks with disruption of mitochondrion enhanced ferroptosis for synergistic immune activation

Lingling Lei[1,2], Zhe Dong[1,2], Li Xu[1], Fengrui Yang[1], Baoli Yin[1], Youjuan Wang[1], Renye Yue[1], Guoqiang Guan[1], Juntao Xu[1], Guosheng Song[1,3,*] and Xiao-bing Zhang[1,3,*]

[1] State Key Laboratory of Chemo/Bio-Sensing and Chemometrics, College of Chemistry and Chemical Engineering, Hunan University, Changsha 410082, P. R. China

[2] These authors contributed equally

[3] Lead Contact

* Correspondence:

*songgs@hnu.edu.cn* (G.S.), *xbzhang@hnu.edu.cn* (X-B.Z.)

**Materials and Characterization.**

Zinc nitrate hexahydrate ($Zn(NO_3)_2 \cdot 6H_2O$), fluorouracil, N, N-Dimethylformamide (DMF), triethylamine (TEA, $(C_2H_5)_3N$)) were purchased from Tianjin Heowns biochemical technology Co., Ltd. PBS (pH 7.4) were obtained from Beijing Solarbio Science & Technology Co., Ltd. Fetal bovine serum (FBS) and 1640 cell culture medium was purchased from Gibco (Grand Island, NY, USA). 2',7'-dichlorofluorescin diacetate (DCFH-DA, > 97%) was purchased from Sigma-Aldrich Co. Ltd. GSH assay kit, acridine orange (AO), and MDA assay kit was purchased from Beijing Solarbio Science & Technology Co., Ltd. Mitochondrial membrane potential assay kit with JC-1, ATP assay kit, phospho-histone $H_2$A.X (Ser139) rabbit monoclonal antibody, immunol fluorence staining kit with Cy3-labeled goat anti-rabbit IgG were purchased from Beyotime Biotechnology Co. Ltd. Anti-Glutathione peroxidase 4 antibody was purchased from Abcam (Cambridge, UK). Liperfluo (LPO) was acquired from Dojindo Laboratorise (Kumamoto, Japan). 3-(4, 5-dimethyl-2-thiazolyl)-2, 5-diphenyl-2-Htetrazolium bromide (MTT) was obtained from Biofrox, Germany. Calreticulin (CRT) and high mobility group box 1 (HMGB1) antibodies were purchased from Cell Signaling Technology. Mouse interleukin-6 (IL-6) ELISA Kit and mouse tumor necrosis factor-α (TNF-α) High Sensitivity ELISA Kit were purchased from MultiSciences. The chemical and biological reagents were bought from commercial suppliers and used as received without further purification. All buffers and solutions were prepared using ultrapure water prepared through a Millipore Milli-Q water purification system (Millipore, USA).

Transmission electron microscopy was performed by JEM-2100 (JEM-2010, JEOL, Japan) with an accelerating voltage of 200 kV. The mean diameter and zeta potential of the as-prepared nanoparticles were measured by the dynamic laser light scattering (DLS, Zetasizer Nano ZS90, Malvern). X-ray photoelectron spectroscopy (XPS) spectra were performed on a K-Alpha+ instrument (Thermo Fisher Scientific, USA). Thermogravimetry analysis (TGA) was conducted on a TGA-50 (SHIMADZU) thermogravimetric analyzer. About 5-10 mg of sample was heated from room temperature to 700 °C at a ramp rate of 5 °C/min in an air flow. Fourier Transform

Infrared Spectroscopy (FTIR) spectra were recorded on a FTIR spectrometer (IR Affinity-1). The absorbance was measured by UV-vis spectrophotometer (UV-1800, Shimadzu) or microplate reader (Spectra Max, iD3). The amount of Zn element was measured by inductively coupled plasma-mass spectrometry (ICP-MS, 8900, Agilent, USA). $^{19}F$ NMR spectra were recorded on a Bruker-400 spectrometer and the samples were dissolved in $H_2O$ (90%) and $D_2O$ (10%). Fluorescence confocal images of live cells were performed through a Nikon A1plus multiphoton laser scanning confocal microscopy. Flow cytometry assay was performed on Beckman coulter flow cytometer. Magnetic resonance imaging was performed with 7 T MRI scanner (Pharma Scan 70/16 US, Bruker). Tissue slice scanning was performed on Pannoramic MIDI microscope (3DHIESTECH, Hungary).

**Methods.**

**Intracellular concentration of $Zn^{2+}$ detection.**

the CT26 cells were cultured in 6-well dish ($2 \times 10^6$ cells / dish) and incubated with (a) PBS; (b) Zn-Fu MNs (100 μg/mL, contain 5-Fu 40 μg/mL and $Zn^{2+}$ 20 μg/mL). After 12 h dark incubation, cells were washed by PBS for three times and collected in centrifuge tube. Then, the cells were dissolved by hydrogen nitrate ($HNO_3$) and hydrogen peroxide solution ($H_2O_2$) ($HNO_3$: $H_2O_2$ = 4:1) for 12h. Finally, samples were diluted in water and detected by ICP-MS.

**Apoptosis and ferroptosis detection.**

For apoptosis and necrosis detection, CT26 cells were seeded in 12-well plates at $0.5\times10^6$ cells per well and then incubated with (a) PBS; (b) 5-Fu (20 μg/mL); (c) Zn $(NO_3)_2$ ($Zn^{2+}$ 10 μg/mL); (d) Zn-Fu MNs (50 μg/mL, contain 5-Fu 20 μg/mL and $Zn^{2+}$ 10 μg/mL), respectively. After 24 h incubation, the all cells were collected and stained with FITC Annexin V/PI apoptosis kit

(Beyotime Biotechnology Co. Ltd) according to manufacturer's instructions. The apoptosis and necrosis were examined by flow cytometry.

For apoptosis and ferroptosis detection, the process of cells treatment was similar with that of apoptosis and necrosis detection. The all cells were collected and stained with Annexin V-mCherry apoptosis detection kit (Beyotime Biotechnology Co. Ltd) and LPO probe Liperfluo, according to manufacturer's instructions, respectively. The apoptosis and ferroptosis were examined by flow cytometry.

**DCs maturation detection.**

Immature murine bone marrow derived dendritic cells (BMDCs) were purchased from the Shenzhen Otwo Biotech Co., Ltd. CT26 cells were seeded in 6-well culture dish with $3 \times 10^6$ cells per well and co-incubated with (a) PBS; (b) Zn-Fu MNs (50 μg/mL, contain 5-Fu 20 μg/mL and $Zn^{2+}$ 10 μg/mL), respectively. After a further incubation of 24 h, the CT26 cells residues were collected via a centrifugation at 4 °C (2500 rpm, 4 min) and co-cultured with BMDCs ($1 \times 10^6$) for 12 h. The BMDCs were stained with anti-CD11c FITC, anti-CD80 PE and anti-CD86 APC (Biolegend, San Diego, CA, USA), and measured by flow cytometry.

**Detection lipid peroxides and GPX4 in tumor tissues**

To evaluate the ferroptosis performances of Zn-Fu MNs, CT26 tumor-bearing Balb/c mice were subcutaneously immunized (near tumors) with injections of (1) PBS; (2) $Zn(NO_3)_2$ (50 μg $Zn^{2+}$ / mouse, 20 μL); (3) 5-Fu (100 μg 5-Fu / mouse, 20 μL); (4) Zn-Fu MNs (250 μg Zn-Fu / mouse, 20 μL, contain $Zn^{2+}$ 50 μg and 5-Fu 100 μg), respectively. The mice were sacrificed and their tumors were collected and sliced at 3rd post treatment:

For evaluating GPX4 down-regulation within tumor: those tumor slices were stained with anti-GPX4 primary antibody at 4 °C for 12 h, with Cy3-conjugated secondary antibody at 37 °C for 2 h, and with DAPI for 15 min, successively. Finally, the tumor slices were observed by the Pannoramic MIDI microscope (DAPI: Ex = 405 nm; Cy3: Ex = 550 nm).

For evaluating lipid peroxides within tumor: those tumor slices were stained with LPO probe Liperfluo for 30 min, and with DAPI for 15 min. Finally, the tumor slices were observed by the Pannoramic MIDI microscope (DAPI: Ex = 405 nm; Liperfluo: Ex = 488 nm).

**For blood biochemistry and complete blood panel analysis.**

To evaluate systematic toxicity, mice (n = 4) were i.t. injected with Zn-Fu MNs (20 µL, 750 µg Zn-Fu / mouse, contain $Zn^{2+}$ 150 µg and 5-Fu 300 µg). At 3 days post injection, those mice were sacrificed and the blood samples (~ 1 mL) were collected for blood biochemistry assay and complete blood panel analysis (Wuhan Service bio Technology Co., Ltd.). Untreated healthy mice were used as the control.

**Supplementary**

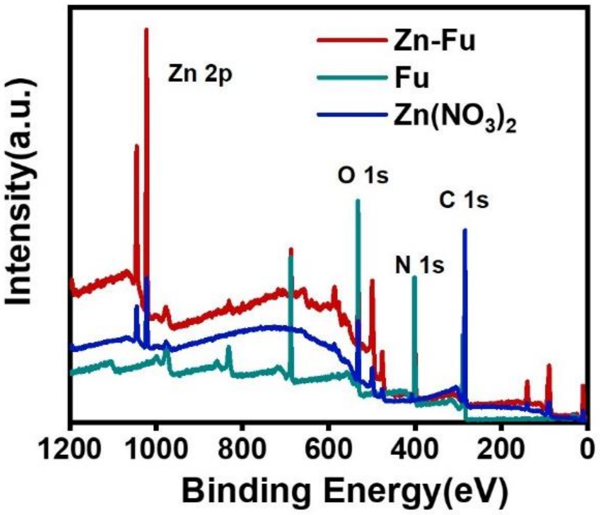


**Figure S1.** XPS spectra of Zn-Fu, Fu and $Zn(NO_3)_2$

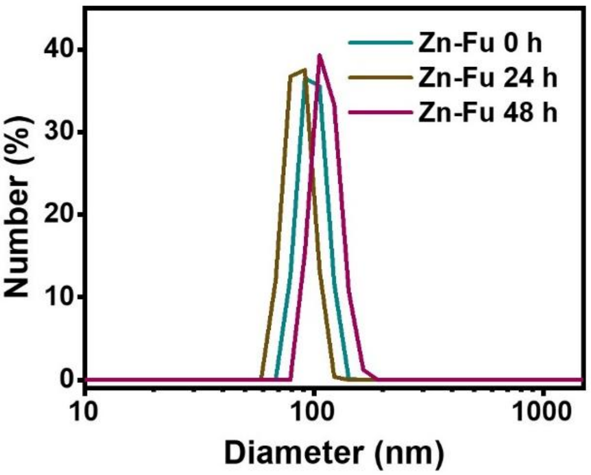


**Figure S2.** DLS profile of Zn-Fu MNs incubated in PBS buffer at different time points.

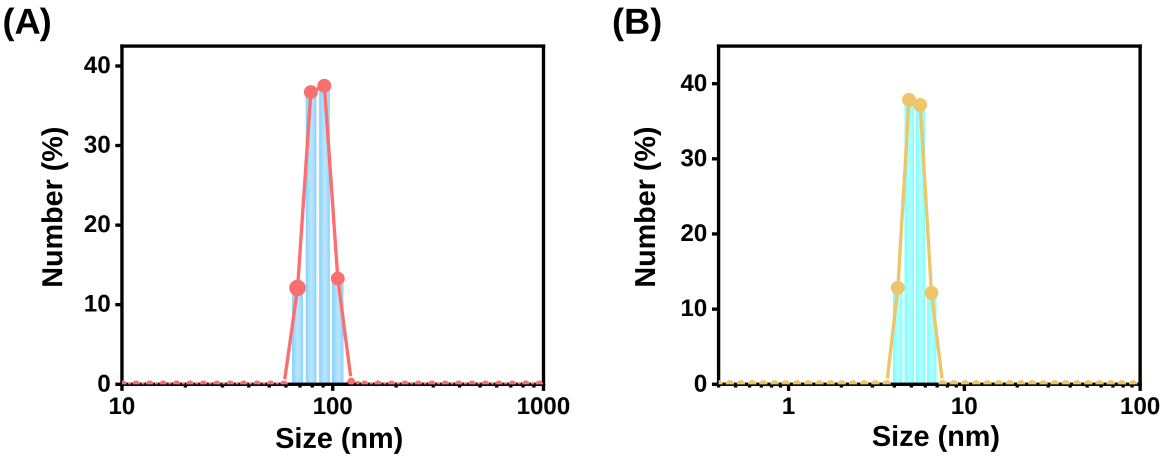


**Figure S3.** The DLS profile of Zn-Fu MNs, before (left) and after (right) treatment in acidic condition.

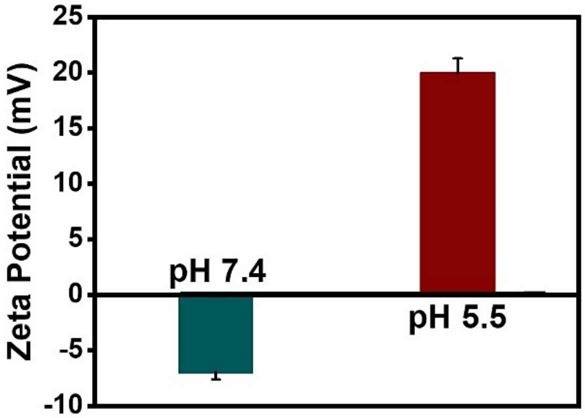


**Figure S4.** Zeta potentials of Zn-Fu MNs at pH 7.4 and pH 5.5 measured by dynamic light scattering (DLS). With the degradation of nanoparticles, the potential changes from negative to positive.

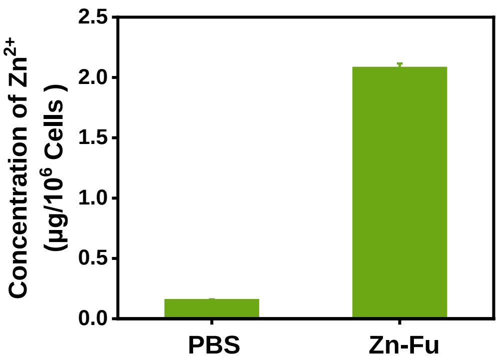


**Figure S5.** Intracellular $Zn^{2+}$ content of cancer cells after PBS or Zn-Fu MNs treatment, detected by ICP-MS.

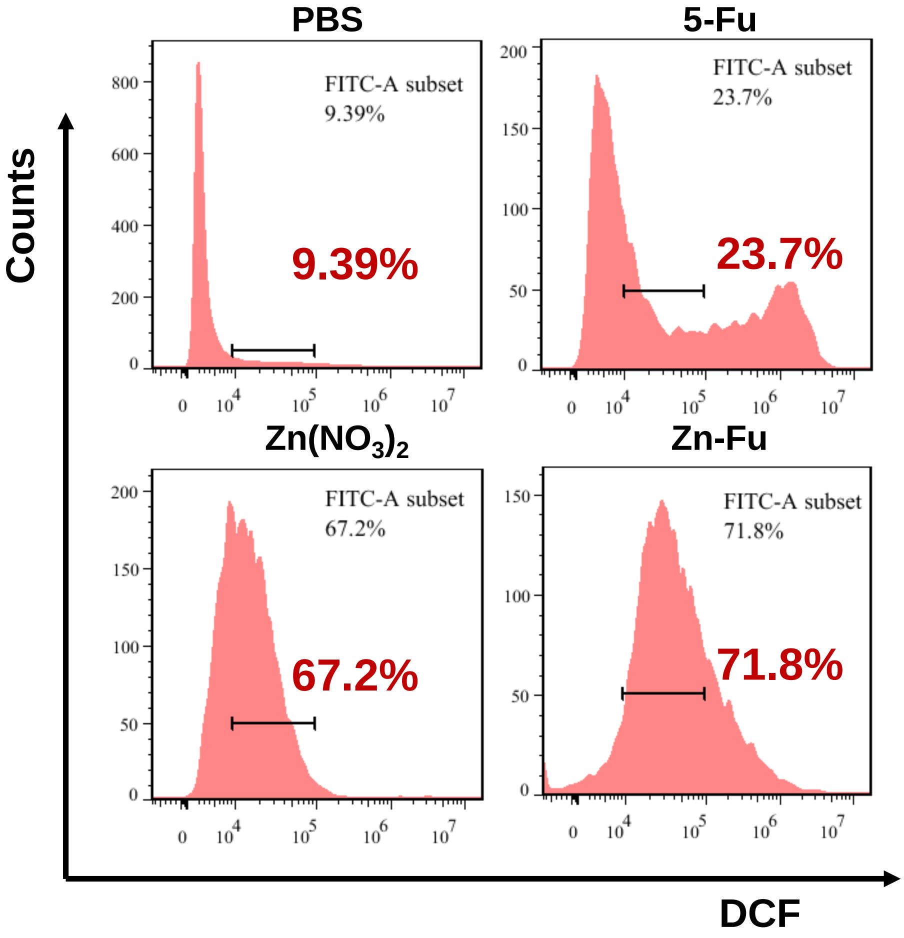


**Figure S6.** A quantitative analysis by flow cytometry about DCF with different treatments [1].

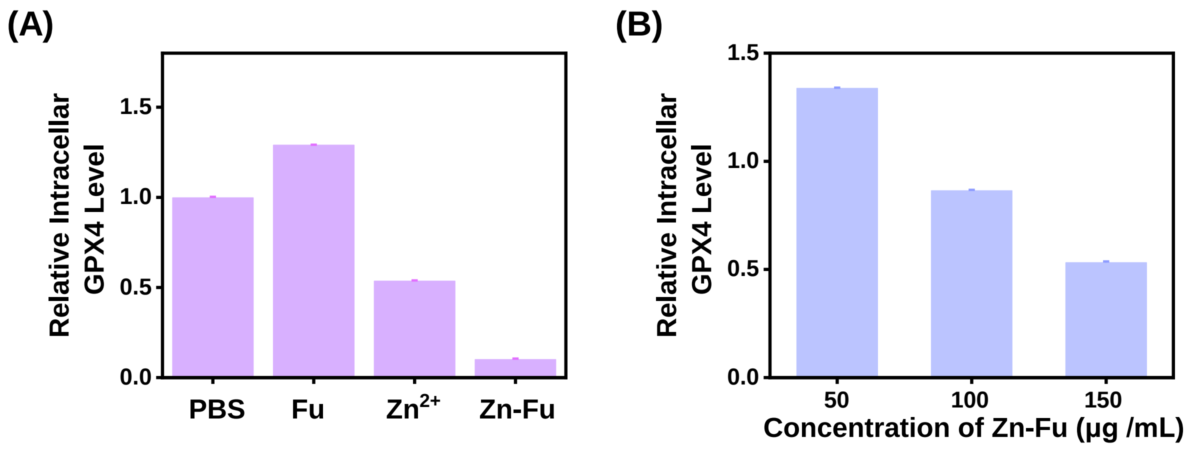


**Figure S7.** The relative intracellular GPX4 level of CT26 cancer cells after different treatments.

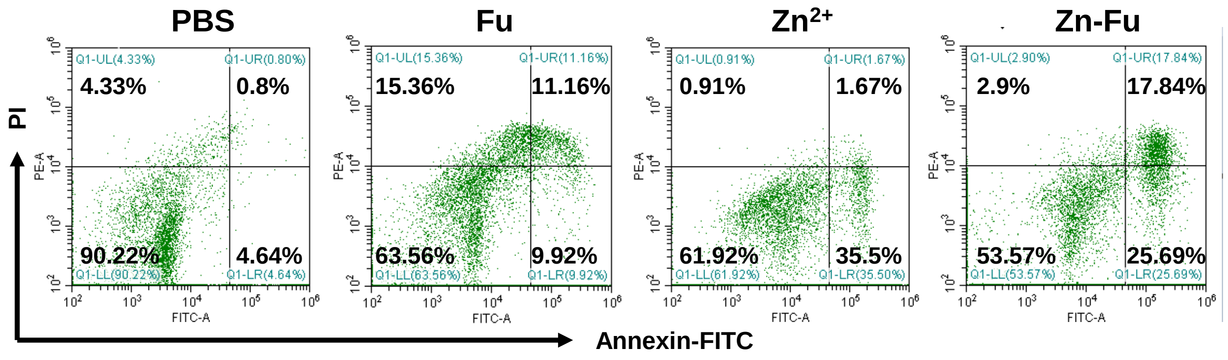


**Figure S8.** Apoptosis level of cancer cells after various treatment, using annexin V/PI as probes for flow cytometry.

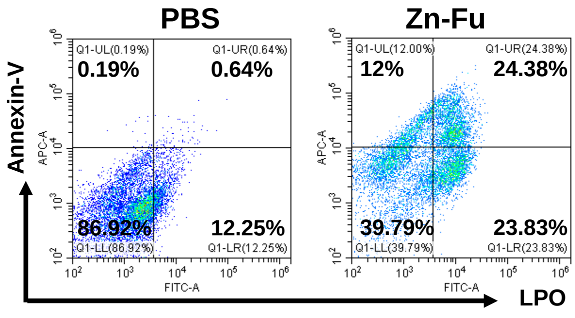


**Figure S9.** Lipid peroxidation level and apoptosis of cancer cells after various treatment, using Liperfluo and annexin V-mCherry as probes for flow cytometry.

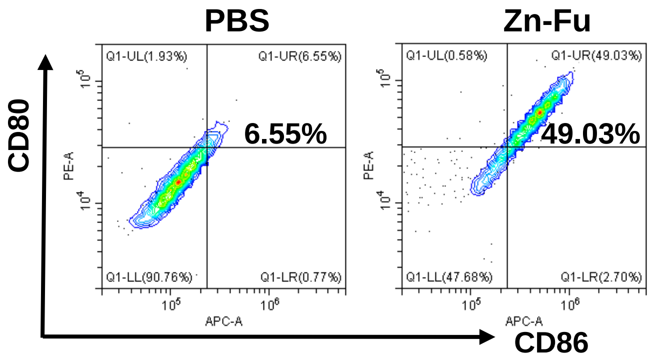


**Figure S10.** Flow cytometric analysis of maturation of DC cells cultured with dying CT26 cells residues induced by PBS or Zn-Fu MNs for 12 h.

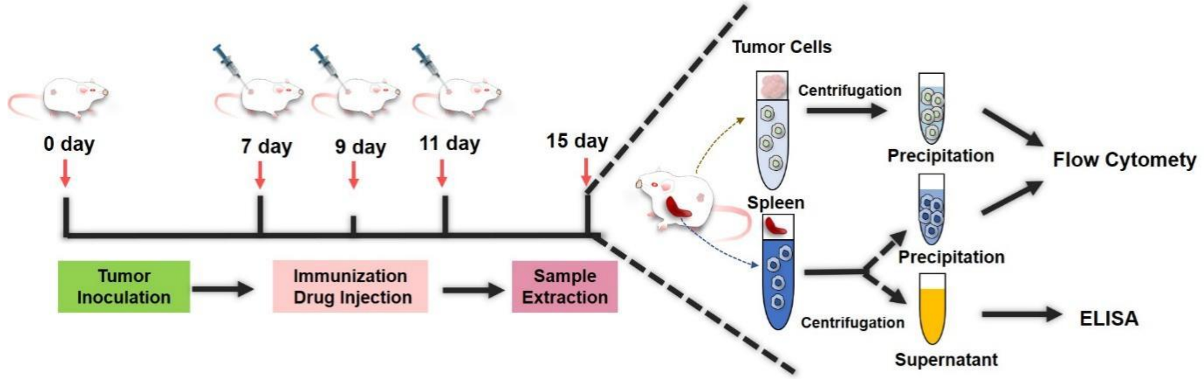


**Figure S11.** Treatment schedule of CT26 tumor-bearing mice treated with different formulations and experimental procedure for the cytokine, DC cells and T cells measurements.

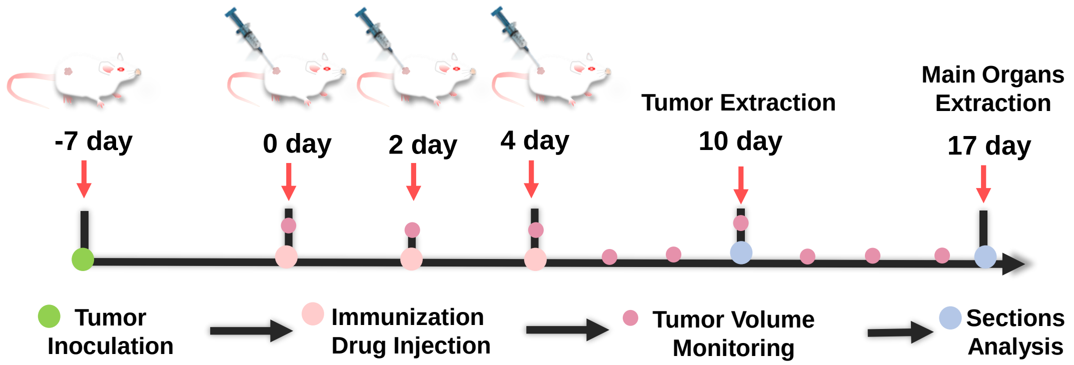


**Figure S12.** Treatment schedule of tumor measurement and safety analysis after CT26 tumor-bearing mice treated with different immunization drugs.

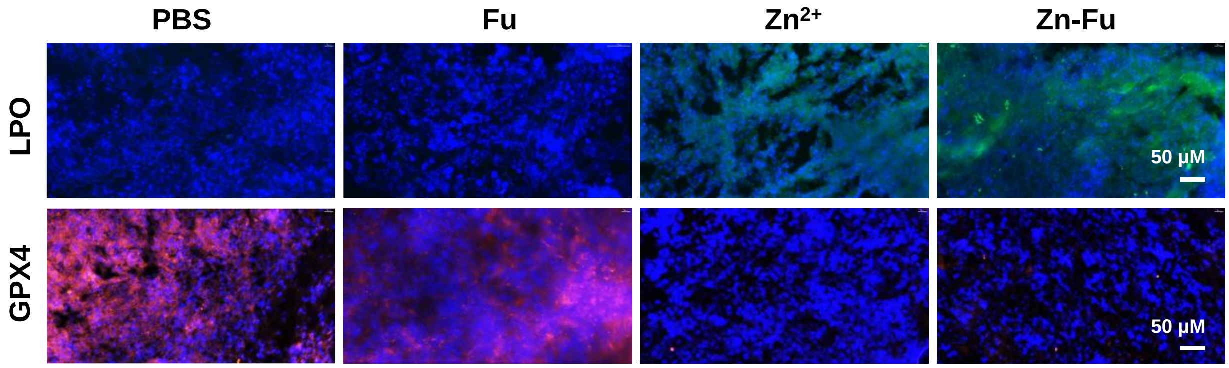


**Figure S13.** LPO and GPX4 stain tumor slice for various treatments. Blue represents DAPI stained nuclei, green is Liperfluo stained oxidized lipids, and red is Cy3 labeled GPX4 antibody.

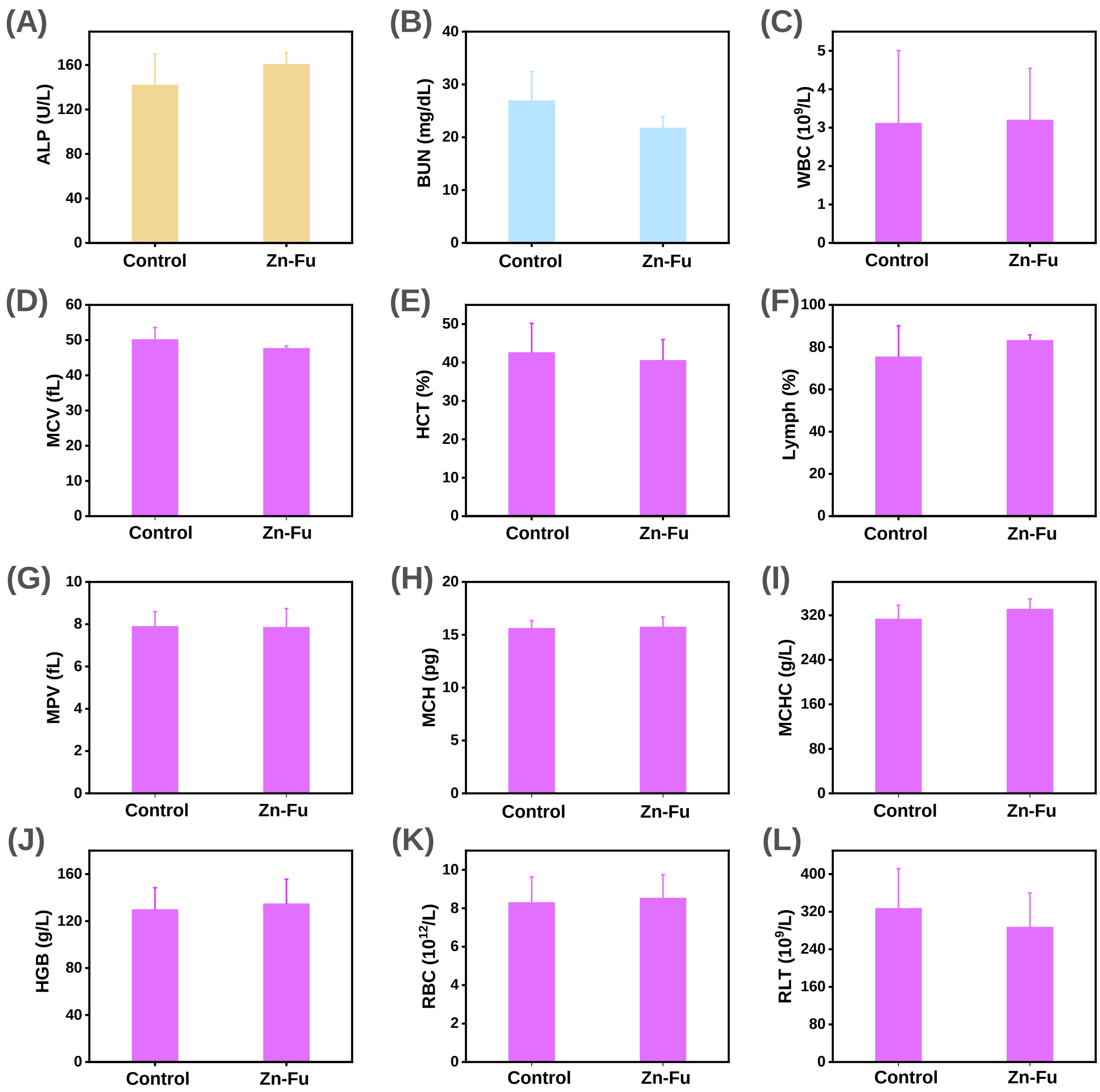


**Figure S14.** Blood biochemistry and complete blood panel analysis of control mice, and mice i. t. injected with Zn-Fu MNs. (A) alkaline phosphatase (ALP); (B) blood urea nitrogen (BUN); (C) white blood cells (WBC); (D) mean corpuscular volume (MCV); (E) hematocrit (HCT); (F) percentage of lymphocytes (Lymph (%); (G) mean platelet volume (MPV); (H) mean corpuscular hemoglobin (MCH); (I) mean corpuscular hemoglobin concentration (MCHC); (J) hemoglobin (HGB); (K) red blood cells (RBC); (L) blood platelet (PLT). The statistic was based on four mice per data point.

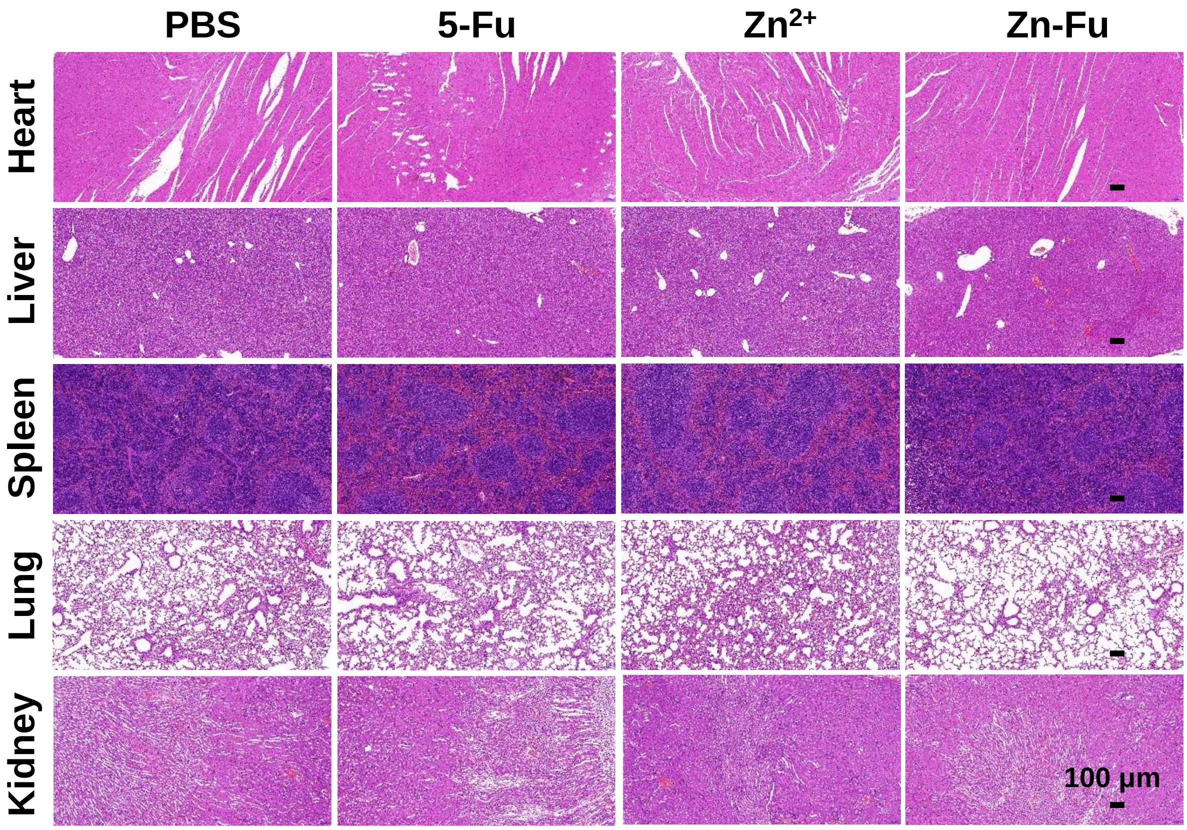


**Figure S15.** H&E-stained tissue sections from mice in each group at the 17th day post first-treatment.

**REFERENCES**

[1] Liu Y, Zhai S, Jiang X, Liu Y, Wang K, Wang C, et al. Intracellular mutual promotion of redox homeostasis regulation and iron metabolism disruption for enduring chemodynamic therapy. Adv Funct Mater. 2021, 31: 2010390.
